# Potential of Triazines as Antidiabetic Agents—A Review of Structures and Pharmacological Activity

**DOI:** 10.3390/ph19071018

**Published:** 2026-06-30

**Authors:** Dorota Łażewska, Diana Strelchuk, Jadwiga Handzlik

**Affiliations:** Chair of Chemical Technology and Biotechnology of Drugs, Faculty of Pharmacy, Jagiellonian University Medical College, ul. Medyczna 9, 30-688 Krakow, Poland; diana.strelchuk.strelchuk@student.uj.edu.pl (D.S.); j.handzlik@uj.edu.pl (J.H.)

**Keywords:** 1,2,4-triazine, 1,3,5-triazine, antidiabetic, fotagliptin, imeglimin

## Abstract

Type 2 diabetes (T2D), a major global health challenge, represents approximately 96% of all cases of diabetes worldwide. Epidemiological forecasts indicate the prevalence of this disease could rise by almost 45% over the next 25 years. T2D is a chronic metabolic disorder characterised by insulin resistance and progressive impairment of β-cell function. Untreated T2D can lead to serious microvascular and macrovascular complications. Traditional therapies have focused primarily on glycaemic control, whereas modern treatment strategies are increasingly centred on the broader pathophysiology of T2D. Among new therapeutic approaches, triazine derivatives have gained significant attention as versatile scaffolds for the development of antidiabetic drugs. This article provides a comprehensive review of triazines (mainly 1,2,4-triazines and 1,3,5-triazines) as promising compounds for the treatment of T2D and its complications. Three databases (Scopus, PubMed, and Web of Science) were searched for the period of 2000–2025. Over the past 25 years, numerous compounds have been described. They were primarily investigated as inhibitors of digestive enzymes and factors that cause diabetic complications. The individual sections discuss the biological activity of these compounds, focusing on SAR analysis and the studies conducted (in vitro, in silico, and in vivo). During this period, two compounds, fotagliptin and imeglimin, have entered clinical use. The results show that triazines have great potential to become antidiabetic drugs. They can not only regulate blood sugar levels (by acting on digestive enzymes, insulin secretion or glucose transport) but also directly prevent serious complications of diabetes.

## 1. Introduction

Diabetes is a metabolic disease of civilisation characterised by elevated blood sugar levels, associated with abnormal insulin secretion and/or action. According to data from the Diabetes Atlas (11th edition, 2025), 589 million people worldwide have diabetes. It is predicted that over the next 25 years, the number of people affected by the disease will increase by around 45% [1]. There are currently a few main types of diabetes: type 1 (insulin-dependent); type 2 (impaired insulin secretion); gestational diabetes, a hybrid form of diabetes; and other forms of diabetes [2]. Diabetes can seriously damage many organs, including the heart, eyes, nerves, blood vessels, and kidneys [3]. Type 2 diabetes (T2D) is the most common form of diabetes, accounting for approximately 96% of all cases worldwide. It is currently one of the leading chronic non-communicable diseases, constituting a serious public health threat. Despite its prevalence, the mechanisms underlying its development remain poorly understood. In T2D, the body’s cells lose their ability to respond properly to insulin (insulin resistance). Initially, this is compensated for by increased insulin secretion. However, over time, the pancreatic β-cells are unable to maintain adequate insulin secretion, leading to insulin deficiency. At the same time, lipids accumulate in non-adipose tissues, disrupting insulin signalling and further exacerbating insulin resistance, particularly in the liver. This results in increased glucose production, reduced glucose uptake, and elevated blood glucose and insulin levels, reinforcing the metabolic vicious circle [4]. Although the causes of T2D are not fully understood, it is believed that an unhealthy lifestyle and metabolic syndrome are the main drivers of elevated triglyceride and non-esterified fatty acid (free fatty acid—FFA) levels, which directly contribute to the development of T2D [5]. An unhealthy lifestyle is associated with an unbalanced diet, a lack of physical activity, and problems with sleep. A few studies have shown a link between sleep and the incidence of diabetes [5,6,7]. In particular, sleep quality, efficiency, and duration are associated with the risk of T2D. In their systematic review, Singh et al. noted a significant connection between sleep deprivation and insulin resistance [6]. Their analysis suggests that adequate sleep is essential for maintaining good metabolic health and preventing complications, such as T2D. Inflammatory markers, such as C-reactive protein (CRP) and serum amyloid A (SAA), and metabolic markers, such as glucagon-like peptide-1 (GLP-1) and non-esterified fatty acid (NEFA) metabolism, influence the link between sleep deficiency and glucose intolerance. Other factors contributing to the development of T2D include being overweight, advanced age, and a family history of diabetes. Diabetes affects the functioning of many organs, leading to serious complications that develop over many years. These complications can be divided into microvascular complications (affecting small blood vessels) and macrovascular complications (affecting large blood vessels) [8].

Currently, the approach to managing T2D has shifted from a model focused solely on lowering blood glucose levels to a more personalised strategy. Now, individual characteristics and needs of each patient, including specific glycaemic and body weight goals, the impact of treatment on weight, the risk of hypoglycaemia, and the prevention of cardiovascular and kidney complications, are taken into consideration.

**Figure 1:**
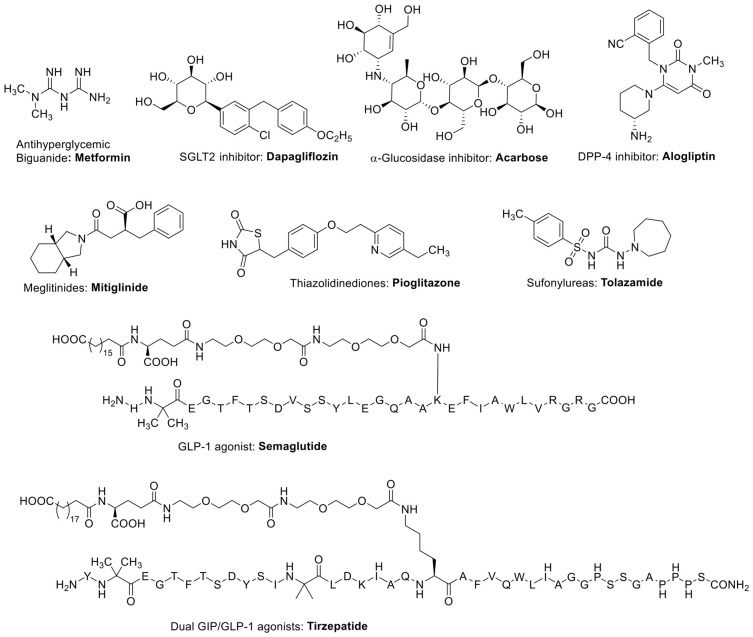
Structures of some drugs used for type 2 diabetes treatment. SGLT2, sodium–glucose cotransporter 2; GLP-1, glucagon-like peptide-1; GIP, glucose-dependent insulinotropic polypeptide; DPP-4, dipeptidyl peptidase 4.

For years, the first-choice drug has been **metformin** (Figure 1), due to its high effectiveness in lowering glucose levels, good safety profile and low cost. For now, to better protect the cardiorenal organs, the use of sodium–glucose transporter 2 (SGLT2) inhibitors, e.g., **dapagliflozin** (Figure 1), and glucagon-like peptide-1 (GLP-1) agonists, e.g., **semaglutide** (Figure 1), is also recommended [9].

**Figure 2:**
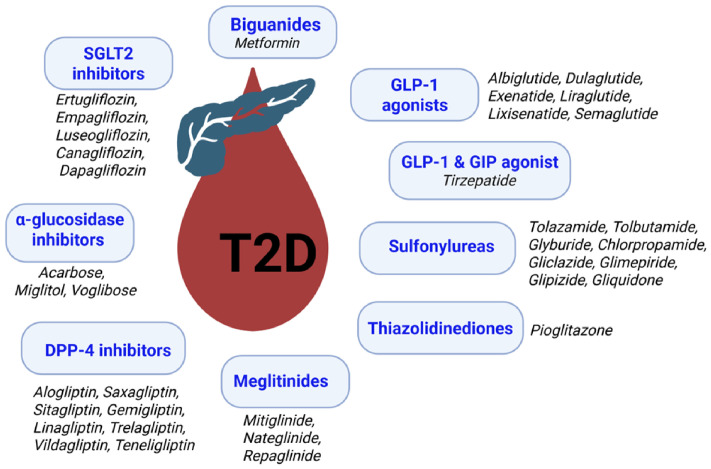
Drugs used to treat type 2 diabetes (T2D). SGLT2, sodium/glucose cotransporter type 2 inhibitors; DPP-4, dipeptidyl peptidase 4; GLP-1, glucagon-like peptide-1; GIP, glucose-dependent insulinotropic peptide. Created in BioRender; Łażewska, D. (2026) https://BioRender.com/rrrfgkc (accessed on 30 May 2026).

Recently, more attention has been paid to **tirzepatide** (Figure 1), a dual agonist of glucose-dependent insulinotropic polypeptide (GIP) and GLP-1 receptor. Tirzepatide can lower blood glucose levels, enhance insulin sensitivity, promote weight loss, and improve lipid metabolism—effects that are particularly important in the management of T2D [10]. Groups of all drugs used for the treatment of T2D are shown in Figure 2 [11], whereas one example drug from each of these is shown in Figure 1.

**Figure 3:**
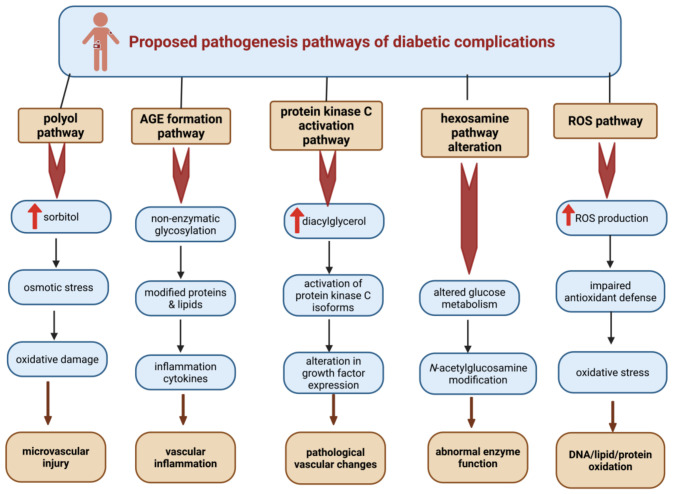
Pathogenesis pathways of diabetes complications. Created in BioRender; Łażewska, D. (2026) https://BioRender.com/6gl80c7 (accessed on 30 May 2026).

Despite the development of numerous antidiabetic drugs, none of them fully meet patients’ needs. Current treatments mainly slow the disease progression. Therefore, scientists are looking for new, safer, and more effective treatments. This research involves searching for new molecules that act on known therapeutic targets and focuses on inhibiting mechanisms that contribute to the development of diabetic complications. Five main pathways resulting in complications have been identified (Figure 3) [12].

In addition, new therapeutic targets are being investigated, e.g., non-steroidal mineralocorticoid receptor antagonists [13], histone deacetylase 4 (HADC-4) inhibitors [14], sirtuin 1 activators [15], and multi-target approaches [16].

**Figure 4:**
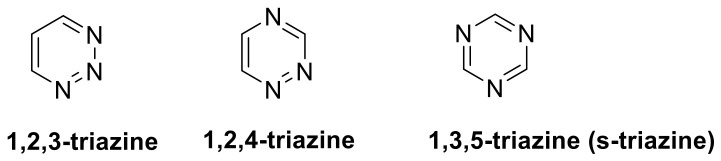
Isomers of triazine rings.

Triazines are an important group of biologically active compounds. There are three isomers of triazines: 1,2,3-triazines; 1,2,4-triazines; and 1,3,5-triazines (Figure 4).

The different positions of nitrogen atoms within the six-membered ring lead to distinct physicochemical properties, metabolic behaviours, and pharmacological activities. Among these three isomers, derivatives of 1,2,3-triazine are the least studied. They rarely occur alone as compounds and are typically found in fused systems with various rings, such as benzene, indole, or pyrazole [17]. In contrast, derivatives of 1,2,4-triazines and 1,3,5-triazines occur both as independent compounds and in fused systems, and they are characterised by a wide range of biological activities (for review, see [18,19]). Among these activities, antidiabetic effects are also included. The first-line drug for T2D, metformin, is itself a derivative of 1,3,5-triazine.

This review provides a summary of the achievements of the past 25 years (2000–2025) in the search for potential antidiabetic drugs among 1,2,3-triazine; 1,2,4-triazine; and 1,3,5-triazine derivatives. Structure–activity relationships (SARs) and mechanisms are discussed, with attention paid to the kinds of biological tests and additional studies performed. For this purpose, three databases (Scopus, PubMed, and Web of Science) were searched using the terms: first “triazine diabetic”; then “1,2,3-triazine diabetic”; “1,2,4-triazine diabetic”; and “1,3,5-triazine diabetic”. The results were verified. Those that were not related to diabetes, were not experimental studies, or did not concern synthesis or information about new compounds were excluded. Some bibliographic references were also excluded due to unavailability. In our review, we focused primarily on new compounds studied during this period, but we also discussed compounds introduced into therapy during this time.

The results were arranged by the type of isomer and divided into three main groups: (I) 1,2,3-triazines; (II) 1,2,4-triazines; and (III) 1,3,5-triazines. Pharmacological activities and structures are discussed below.

## 2. 1,2,3-Triazines as Antidiabetic Agents

### 1,2,3-Triazines as α-Glucosidase Inhibitors

α-Glucosidase is found in the cells of the small intestine and is responsible for breaking down disaccharides and oligosaccharides into absorbable monosaccharides. α-Glucosidase inhibitors slow carbohydrate digestion and glucose absorption, reducing blood glucose levels after a meal. Commonly used medicines in this group may cause gastrointestinal side effects, such as bloating, diarrhoea, or abdominal pain.

**Figure 5:**
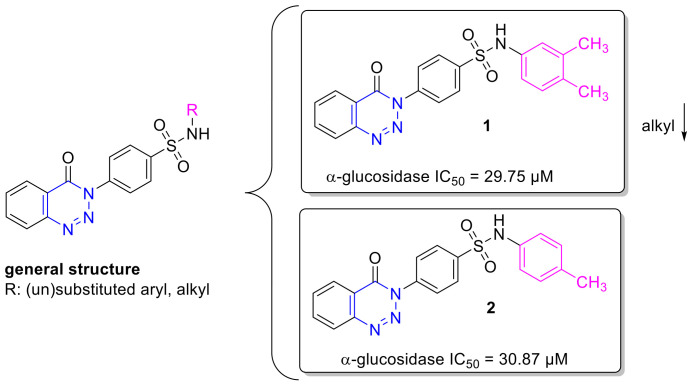
Structures of α-glucosidase inhibitors, as described by Khalid et al. [20].

Khalid et al. described chemical synthesis and biological evaluation of novel 1,2,3-benzotriazin-4(3H)-one derivatives as α-glucosidase inhibitors [20]. **Thirteen** new compounds with a sulfonamide moiety were obtained under mild conditions (Figure 5). α-Glucosidase inhibitory activity was evaluated in an in vitro spectrophotometric assay, with α-glucosidase from *Saccharomyces cerevisiae* and p-nitrophenyl-α-D-glucopyranoside as the substrate. The majority of compounds had stronger inhibitory activity than the drug acarbose (Figure 1; IC_50_ = 37.38 μM). Structure–activity relationship (SAR) analysis showed that aryl sulfonamides were more effective inhibitors than alkyl derivatives. The structures of the two most potent compounds **1** and **2** are shown in Figure 5. Molecular docking studies were performed using a homology model of yeast α-glucosidase to understand how these compounds bind to the enzyme’s active site. Aryl sulfonamides form more complex and stabilising interactions within the enzyme’s binding cavity.

## 3. 1,2,4-Triazines as Antidiabetic Agents

### 3.1. 1,2,4-Triazines as Monotarget Ligands

#### 3.1.1. 1,2,4-Triazines as α-Glucosidase Inhibitors

1,2,4-triazines as α-glucosidase inhibitors were described by Wang et al. [21] and Valipour et al. [22]. In both analysed studies, α-glucosidase inhibitory activity was determined using an in vitro spectrophotometric assay, with α-glucosidase from *Saccharomyces cerevisiae* and p-nitrophenyl-α-D-glucopyranoside as the substrate. The release of *para*-nitrophenol was monitored at 405 nm, and IC_50_ values were calculated relative to acarbose, the reference inhibitor (Figure 1).

**Figure 6:**
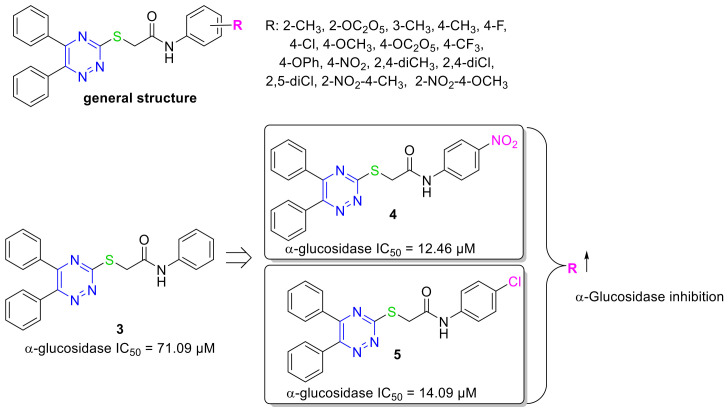
Structures of α-glucosidase inhibitors, as described by Wang et al. [21].

Wang et al. investigated a series of 2-((5,6-diphenyl-1,2,4-triazin-3-yl)thio)-*N*-arylacetamides with a thioether–acetamide bridge (general structure; Figure 6) [21].

**Figure 7:**
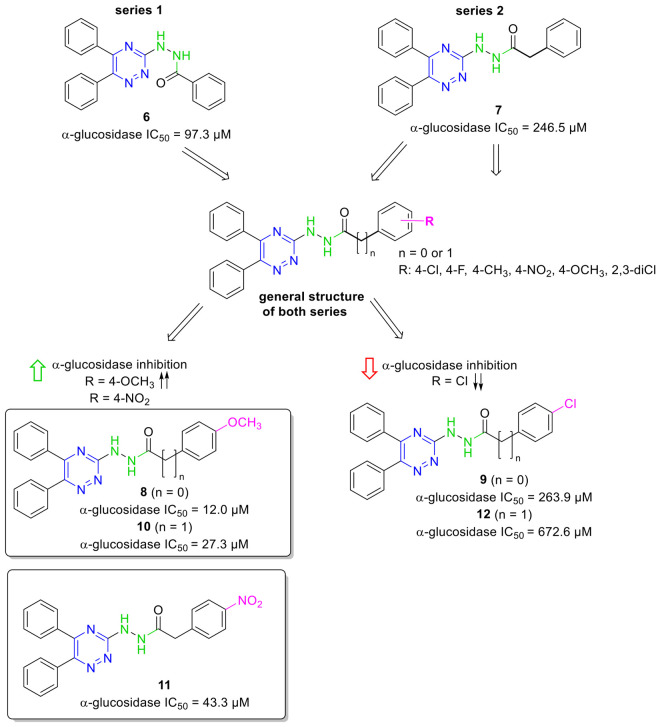
Structures of α-glucosidase inhibitors, as described by Valipour et al. [22].

**Seventeen** compounds were obtained by condensation of 5,6-diphenyl-1,2,4-triazine-3-thiol with appropriate 2-chloro-*N*-arylacetamides. All compounds tested in vitro for α-glucosidase inhibitory activity inhibited the enzyme in the micromolar range (<100 μM). Each introduced substituent showed better or comparable activity (R: 2-methyl; IC_50_ = 72.68 μM) with the unsubstituted derivative (Figure 7; compound), much better than the reference acarbose (Figure 1) (IC_50_ = 817.38 μM). Among this series, two compounds **4** (4-NO_2_ substituent) and **5** (4-Cl substituent) (Figure 7) exhibited the highest inhibitory activities, with an IC_50_ of 12.46 μM and 14.09 μM, respectively. Docking studies were done using the homology model built on the crystal structure of isomaltase from Saccharomyces cerevisiae (PDB: 3AJ7). The results demonstrated that the nitro group (compound **4**) formed an additional hydrogen bond with Arg-312, leading to enhanced binding affinity. Similarly, compound **5**, containing a *para*-chloro substituent, showed strong inhibition, attributed to a stabilising Cl–π interaction with His-239. In this series, electron-withdrawing substituents significantly improved activity.

Valipour et al. designed two series of 3- hydrazide-1,2,4-triazine derivatives based on the 5,6-diphenyl-1,2,4-triazine scaffold (Figure 7) [22]. **Ten** compounds (four in the first series; six in the second series) with a carbohydrazide linker at position three of the triazine ring were synthesised in a four-step synthesis. All of these tested in vitro exhibited α-glucosidase inhibitory activity superior to acarbose (IC_50_ = 752 ± 20 μM). Within the first series, compound **8** (Figure 7), bearing a *para*-methoxybenzohydrazide moiety, emerged as the most potent inhibitor (IC_50_ = 12.0 ± 0.4 μM), demonstrating approximately 60-fold greater activity than acarbose. In contrast, compound **9** (Figure 7), containing a *para*-chloro substituent, showed markedly reduced potency (IC_50_ = 263.9 ± 17.0 μM), suggesting that halogen substitution within the rigid benzohydrazide framework is unfavourable for enzyme binding. As was further confirmed in the second series, compound **10** (with *para*-methoxy substituent) was the most potent inhibitor (IC_50_ = 23.7 ± 1.2 μM), whereas compound **12** (with *para*-chloro substituent) was the weakest. In this series, compound **11** (*para*-nitro group) has an IC_50_ of 43.1 ± 1.3 μM. SAR analysis indicated that benzohydrazide derivatives were generally more potent than their phenylacetohydrazide counterparts and that *para*-methoxy substitution significantly enhanced inhibitory activity. Molecular docking studies performed on the most active derivative **8** (PDB: 7P2Z) revealed multiple stabilising interactions within the catalytic pocket of α-glucosidase, particularly with Asp282, Trp481, and Asp616, which are essential for substrate recognition and catalytic stabilisation. The hydrazide linker and triazine core were identified as key structural elements responsible for hydrogen bonding and π–π interactions within the active site. Enzyme kinetic studies confirmed a competitive inhibition mechanism (K_i_ ≈ 12 μM), indicating that compound **8** binds directly to the catalytic site. Notably, compounds **8** and **11** were further evaluated in cytotoxicity assays (MTT test on HCT-116, MDA-MB-231, and A549 cell lines). Compound **8** exhibited low cytotoxicity, with IC_50_ values of 180.7 ± 6.3 μM, 163.8 ± 5.2 μM, and 175.2 ± 7.3 μM against HCT-116, MDA-MB-231, and A549 cells, respectively. Compound **11** also showed a favourable safety profile, with IC_50_ values of 112.1 ± 4.7 μM, 116.7 ± 3.8 μM, and 126.2 ± 5.7 μM against the same cell lines. Furthermore, in an in vivo blood glucose determination test in mice, compound **8** demonstrated significant hypoglycaemic activity comparable to acarbose, thereby providing broader pharmacological validation of the hydrazide series.

#### 3.1.2. 1,2,4-Triazines as Inhibitors of Advanced Glycation End Products

**Figure 8:**
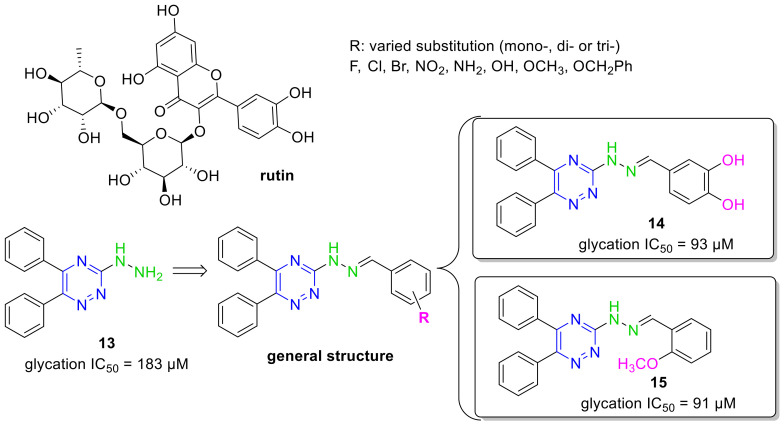
Structures of selected 1,2,4-triazine derivatives active against AGE formation and AGE-induced inflammatory signalling in monocytes, as described by Jahan et al. [23].

Jahan et al. described a series of diphenyl-1,2,4-triazine derivatives as potential inhibitors of the formation of advanced glycation end products (AGEs) and of inflammatory signalling associated with diabetic complications [23]. AGEs are formed through non-enzymatic reactions between reducing sugars and proteins (the Maillard reaction). This is a physiological process, and AGEs are naturally produced in the body during metabolism, but they can also be introduced through processed foods exposed to high temperatures. Excess accumulation of AGEs may disrupt cellular signalling by altering protein structures and functions, generating reactive oxygen species (ROS) and inflammatory mediators, and interacting with AGE-specific receptors (RAGE). As a result, AGEs accumulation contributes to many lifestyle-related diseases, including diabetes [24,25]. Jahan et al. evaluated the antiglycation activity of compounds previously described by Shamin et al. as dual inhibitors of α-amylase and α-glucosidase (see Section 3.2.1) [26]. Of the **twenty-six** compounds tested, nine showed inhibitory activity greater than 50%, and their IC_50_ values were determined. These compounds demonstrated strong inhibition of methylglyoxal (MGO)-induced AGE formation (in a fluorescence-based assay), with IC_50_ values in the range of 91–259 μM. Among these were an unsubstituted hydrazine derivative, compound **13** (IC_50_ = 183 ± 0.02 μM; Figure 8), and compounds **14** and **15** (Figure 8). Compounds **14** and **15** showed the highest inhibitory activity (IC_50_ values of 91 ± 0.04 μM and 93 ± 0.02 μM, respectively) in the whole series, significantly outperforming the reference inhibitor rutin (IC_50_ = 180 ± 0.08 μM; Figure 8). SAR analysis showed that the diphenyl-1,2,4-triazine scaffold itself played an important role in antiglycation activity. The type and position of substituents on the phenyl ring directly connected to the hydrazine group also influenced this activity.

The presence of hydroxyl or alkoxyl groups (e.g., the methoxyl group in compound **15**) increases the degree to which AGE formation is inhibited.

The most active derivatives (**14** and **15**) were further evaluated in cellular models relevant to diabetic inflammation. Cytotoxicity of compounds was determined using MTT assay in hepatocytes (HepG2) and WST-1 assay in monocytes (THP-1), demonstrating low cytotoxicity (<30%) even at the highest concentration of 100 μM. Further studies on AGE-stimulated THP-1 monocytes revealed that the tested compounds significantly reduced intracellular oxidative stress by the fluorescence assay with 2,7-dichlorodihydrofluorescein diacetate (DCFH-DA). Immunocytochemistry and Western blot analyses showed inhibition of NF-κB and p38 MAPK activation and downregulation of RAGE and COX-2 expression, while ELISA assays confirmed decreased prostaglandin E2 (PGE_2_) production. SAR analysis suggested that the antiglycation activity is connected with the hydrazine moiety and hydroxyl substituents.

Overall, a few compounds had strong inhibition of AGE formation, low cytotoxicity, and effective suppression of inflammatory signalling in monocytes, highlighting their potential as multifunctional agents for preventing inflammation-related diabetic complications [23].

#### 3.1.3. 1,2,4-Triazines as Aldose Reductase Inhibitors

Aldose reductase (ALR) is an important enzyme in the polyol pathway. The polyol pathway plays a significant role in the development of diabetic complications. It involves the conversion of glucose to sorbitol (via ALR2 with NADPH), and subsequently to fructose (via sorbitol dehydrogenase). Under hyperglycaemic conditions, ALR2 activity increases, leading to excessive sorbitol accumulation. This process consumes NADPH, reduces glutathione levels, and enhances oxidative stress. Sorbitol buildup also causes osmotic stress. As a result, cellular damage and diabetic complications occur (including neuropathy, nephropathy, retinopathy, and cataract formation); therefore, ALR2 and the polyol pathway are potential therapeutic targets for T2D. Although many ALR2 inhibitors have been developed, none have received FDA approval, mainly due to poor selectivity over ALR1, which can cause toxic side effects [12,27].

**Figure 9:**
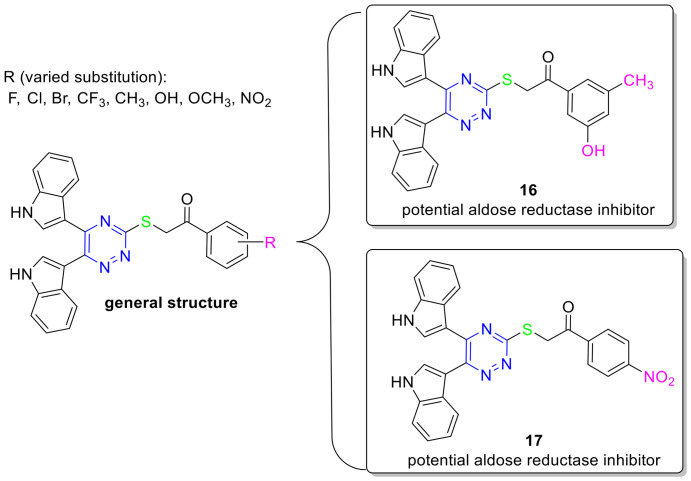
Potential aldose reductase inhibitors from in silico studies, as described by Roney et al. [28].

Roney et al. investigated, by in silico methods, a series of **twelve** bis-indole-1,2,4-triazines as potential antidiabetic agents targeting human aldose reductase (hALR) [28]. These compounds were previously described by Khan et al. as ß-glucuronidase inhibitors with anticancer and antibacterial activity (see Section 3.2.2) [29]. In this study, the authors first confirmed, based on differential expression analysis, that hALR encoding gene AKR1B1 is overexpressed in diabetic patients (Log2FC = 0.62, *p* < 0.01). Then, they conducted molecular docking, MM/GBSA calculations, molecular dynamics (MD) simulations, principal component analysis, and post-MD free binding energy calculations. Molecular docking was done on the human protein (PDB: 1US0). Among the evaluated compounds, compound **16** (Figure 9) demonstrated the strongest docking affinity (−62.12 kcal/mol) and a post-MD binding free energy of −54.93 ± 3.92 kcal/mol, whereas compound **17** (Figure 9) exhibited the most favourable MM/GBSA score (−85.97 kcal/mol), with a post-MD binding free energy of −50.00 ± 3.09 kcal/mol. Both compounds were identified as lead candidates for further development of an antidiabetic drug. However, in vitro and in vivo studies are required to validate their therapeutic potential.

#### 3.1.4. 1,2,4-Triazines as Sodium-Glucose Cotransporter 2 Inhibitors

Sodium–glucose cotransporter 2 (SGLT2) is a transporter protein found in the kidneys that reabsorbs ninety per cent of glucose. A single glucose molecule, together with one sodium cation, is transported by secondary active transport in accordance with the sodium gradient generated by the Na+/K+ ATPase. Subsequently, by diffusion, the glucose molecule is transported from the interior of the cell by the GLUT2 (glucose transporter 2) carrier. The remaining 10% of glucose is reabsorbed similarly by SGLT1 (1 glucose molecule per 2 sodium cations) and GLUT1 [30].

SGLT2 inhibitors help the kidneys to excrete excess glucose, thereby lowering plasma glucose levels. Moreover, they lead to weight loss, reduce the risk of cardiovascular disease, and protect the kidneys. Twelve drugs from this group have been introduced into medical practice since 2012. The first of this group was dapagliflozin (Figure 1), which entered the market in 2012 [31].

**Figure 10:**
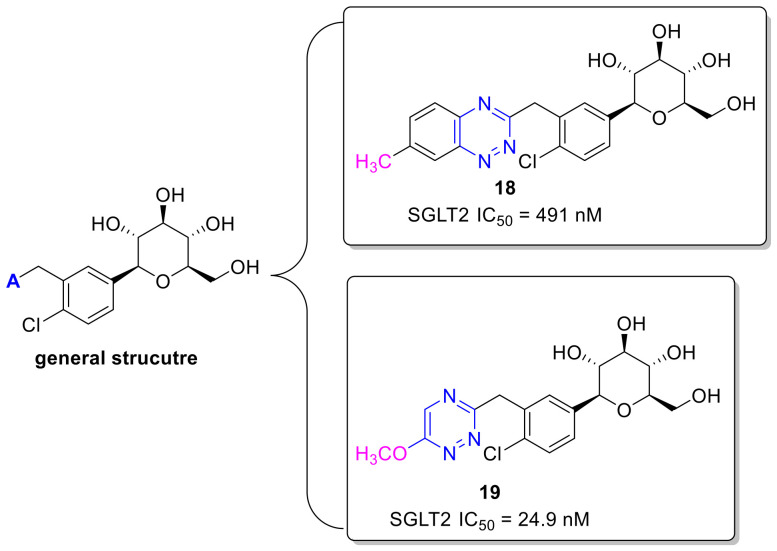
Structures of sodium–glucose cotransporter 2 (SGLT2) inhibitors, as described by Kang et al. [32].

In 2011, Kang et al. described **two** glucoside derivatives with 1,2,4-triazine as SGLT2 inhibitors for the treatment of T2D [32]. The design strategy was based on structural modification of dapagliflozin (Figure 1) by replacing its distal aromatic ring with heterocyclic triazine-containing motifs. The authors hypothesised that incorporating a nitrogen-containing heteroaromatic system could improve the physicochemical properties of the molecules while maintaining biological activity (Figure 10).

To evaluate the biological activity of the synthesised compounds, an in vitro cell-based SGLT2 inhibition assay was performed using Chinese hamster ovary (CHO) cells stably transfected with human SGLT2. Inhibitory potency was determined by measuring the uptake of radiolabelled ^14^C-α-methyl-D-glucopyranoside, a glucose analogue transported by SGLT2. Dapagliflozin, used as the reference compound, displayed excellent inhibitory potency toward human SGLT2, with an IC_50_ of 0.49 nM, confirming its strong affinity for the transporter and serving as the benchmark for comparison.

Compound **18** (Figure 10), containing a fused benzotriazine ring, exhibited significantly reduced inhibitory activity, with an IC_50_ value of 491 nM. This substantial loss in potency indicated that the rigid, planar bicyclic benzotriazine scaffold is poorly tolerated within the SGLT2 binding pocket, likely due to steric or conformational limitations. Compound **19** (Figure 10), featuring a monocyclic 1,2,4-triazine ring with a methoxy substituent, had an IC_50_ of 24.9 nM. Although its inhibitory potency is lower than that of dapagliflozin, the monocyclic triazine ring is substantially better tolerated than the fused bicyclic benzotriazine moiety; it can represent a viable scaffold for further structural optimisation. The authors did not show structures of other derivatives but confirmed that other compounds in this group showed IC_50_ values in the range of 24.9–833 nM. These structures and other compounds, obtained by the Green Cross Cooperation as SGLT2 inhibitors, received patent protection [33].

#### 3.1.5. 1,2,4-Triazines as Dipeptyl Peptidase-4 Inhibitors

**Figure 11:**
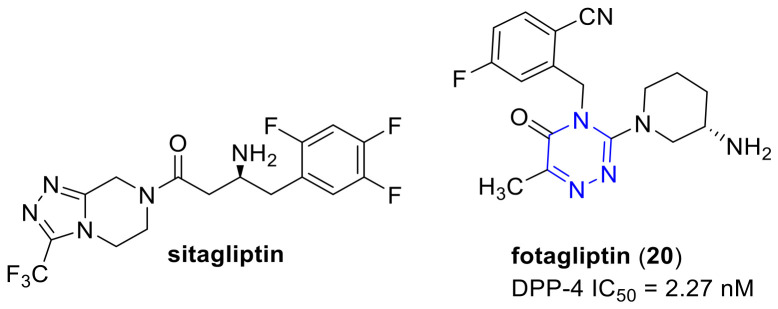
Structures and activities of sitagliptin and **fotagliptin**.

Dipeptyl Peptidase-4 (DPP-4) is an enzyme that degrades incretin hormones, such as glucagon-like peptide-1 (GLP1) and glucose-dependent insulinotropic peptide (GIP). A few recent reviews describe the search for new ligands and the clinical utility of currently available DPP-4 drugs [33,34,35]. DPP-4 inhibitors (gliptins) have been available for T2D treatment since 2006, when sitagliptin (Figure 11) was approved by the FDA. Nowadays, available drugs bind reversibly to the catalytic site of DPP-4 and generally do not interfere with its non-enzymatic physiological functions [34,35,36].

**Fotagliptin** (compound **20**; Figure 11) is a new DPP-4 inhibitor developed in China for T2D. Fotagliptin increases the concentration of active incretin hormones, primarily GLP-1 and GIP, which in turn enhances glucose-dependent insulin secretion and decreases glucagon release. In recent years, four studies have been published presenting the results of clinical trials that, among others, led to the approval of this compound as a drug [37,38,39,40]. Ding et al. described [37] pharmacokinetic interaction and safety of fotagliptin (24 mg daily) in monotherapy and combination with metformin (2 × 1000 mg daily) for 8 days of use. In a trial, eighteen healthy males participated. The results showed that monotherapy and combined therapy were well-tolerated. No serious adverse events and episodes of hypoglycaemia were reported. Furthermore, combined therapy did not cause drug–drug interactions. Thus, fotagliptin is an attractive therapeutic option for patients failing metformin monotherapy. Wu et al. [38] described results from the phase Ib study. This study evaluated the safety, pharmacokinetics, and pharmacodynamics of fotagliptin benzoate in 14 Chinese patients with T2D. For 14 days, patients received either a 24 mg dose of the drug once daily or a placebo. Pharmacokinetic data showed rapid absorption following oral administration. Steady-state plasma concentration was safely achieved by day 12. An approximate 10-fold increase in GLP-1 levels was observed compared with the placebo. The drug was well-tolerated, causing no serious adverse events or episodes of hypoglycaemia. However, further studies are necessary. Xu et al. [39] reported results from a phase III trial evaluating the efficacy and safety of fotagliptin monotherapy in patients with T2D. It was a multi-centre, randomised, double-blind, placebo-controlled trial involving 458 participants. Patients were randomly assigned in a 2:1:1 ratio to groups receiving fotagliptin (12 mg daily), alogliptin (25 mg daily), or placebo. The double-blind period lasted for 24 weeks. The total follow-up period was 52 weeks. At week 24, fotagliptin significantly reduced the mean glycated haemoglobin (HbA1c) level by 0.70%, demonstrating a clinical advantage over placebo. The safety profile was favourable. The incidence of hypoglycaemia was 1.0% in the fotagliptin group. No serious drug-related adverse events occurred, and no significant effect on body weight was observed. It is as effective as alogliptin. Yu et al. presented the results of a phase III clinical trial conducted in China, which aimed to evaluate the efficacy and safety of fotagliptin in combination with metformin for the treatment of T2D [40]. The trial focused on patients who were unable to maintain normal blood glucose levels with metformin monotherapy alone. A total of 408 participants were randomly assigned to receive fotagliptin or placebo (in a 2:1 ratio) as part of a 24-week double-blind treatment. Patients received a fixed dose of metformin (>1500 mg daily). During the 24-week double-blind period, participants receiving fotagliptin (12 mg) showed a significant reduction in HbA1c levels compared with those receiving placebo. Fotagliptin was well-tolerated, had a low risk of hypoglycaemia, and had no significant effect on body weight. Long-term data from a subsequent 52-week open-label phase further confirmed the drug’s sustained efficacy and safety profile.

To summarise, clinical trials with fotagliptin have demonstrated a significant reduction in HbA1c levels and improved glycaemic control. Furthermore, fotagliptin has demonstrated a favourable safety profile, characterised by a low frequency of hypoglycaemia and serious adverse events. In 2024, fotagliptin entered the market in China [41].

#### 3.1.6. 1,2,4-Triazine as Glucagon-like Peptide-1 Receptor Agonists

The glucagon-like peptide-1 receptor (GLP-1R), which belongs to the GPCR family, regulates glucose and lipid metabolism by binding to the incretin hormone, glucagon-like peptide-1 (GLP-1). GLP-1 is derived from proglucagon and is produced mainly by L-cells in the intestine, pancreatic α-cells, and neurons in the nucleus of the solitary tract. GLP-1 receptor agonists (GLP-1RAs) increase insulin secretion, inhibit glucagon release, delay gastric emptying, and reduce appetite, thereby improving glycaemic control and metabolic health [42].

**Figure 12:**
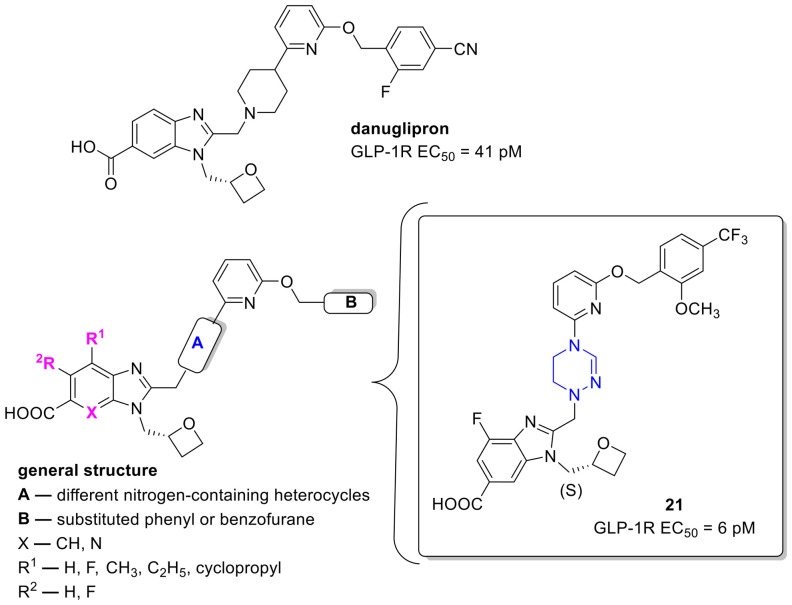
Structures of danuglipron and the most potent glucagon-like peptide-1 receptor agonist, as described by Chen et al. [43].

In 2023, Chen et al. described a series of **forty-one** 5,6-dihydro-1,2,4-triazines as GLP-1RAs [43]. Compounds were obtained after structural modifications of danuglipron (Figure 12). Danuglipron (PF-06882961) is a promising GLP-1RA (found by Pfizer) that was evaluated in clinical trials (phase III) as a potential therapy for T2D [44]. It acts as a full agonist for cAMP signalling and a partial agonist for β-arrestin recruitment, calcium mobilisation, and ERK1/2 phosphorylation. In April 2025, Pfizer announced a discontinuation of the work on danuglipron. The reason was potential drug-induced liver damage in one participant, which resolved after danuglipron was discontinued [45]. Chen et al. rationally introduced three types of structural modifications to danuglipron (Figure 12).

At each stage, compounds with a strong ability to stimulate the GLP-1R receptor were obtained. The activity of the compounds was assessed in HEK293 cells expressing the human GLP-1R receptor by measuring their effect on cAMP accumulation. The potency of the compounds was expressed as EC_50_ values. As a result of this work, compound **21** (Figure 12) was obtained, which demonstrated a seven-fold increase in potency compared with danuglipron (EC_50_ = 6 pM compared with 41 pM). Compound **21** acts as a full agonist in the Gs (cAMP) pathway but remains a partial agonist for ß-arrestin recruitment and calcium mobilisation. This signalling profile was similar to danuglipron but with 10-fold higher potency in secondary pathways. Furthermore, selectivity assays confirmed that compound **21** did not activate other class B GPCR members, including GIPR, GLP-2R, and GCGR. A pharmacokinetic profile of compound **21** was evaluated in SD rats after administration of 5 mg/kg p.o. Compound **21** exhibited a relatively short half-life of 1.05 h and moderate plasma exposure, with a C_max_ of 130 ng/mL and an AUC_last_ of 70 h·ng/mL. Furthermore, in mice with a transgenic hGLP-1R gene, this compound induced a prolonged glucose-lowering effect and greater appetite suppression compared with danuglipron. To fully understand the binding mechanism of 5,6-dihydro-1,2,4-triazines, molecular docking simulations were carried out for compound **21** using the GLP-1R structure obtained by cryo-electron microscopy (PDB ID: 6X1A). The results showed that this compound maintained key polar interactions with residues K197, R380, and Q234. Furthermore, the introduction of a fluorine atom and a methoxy group into compound **21** facilitated additional hydrophobic interactions with the surrounding amino acid residues, which explains its increased potency compared with danuglipron.

### 3.2. 1,2,4-Triazines as Dual or Multi-Target Ligands

#### 3.2.1. 1,2,4-Triazines as Dual α-Amylase and α-Glucosidase Inhibitors

α-Amylase and α-glucosidase are key enzymes in the digestion of dietary starch, catalysing the hydrolysis of polysaccharides and disaccharides into simple glucose molecules. Inhibition of the activity of both enzymes reduced postprandial hyperglycaemia (PPH), a condition characterised by elevated blood glucose levels after eating. Drugs used to lower PPH, such as acarbose, voglibose, and miglitol, are often associated with side effects including diarrhoea, abdominal pain, and flatulence. Due to this significant issue, their use is limited, and the search for new drugs with similar pharmacological activity but different chemical structure is needed [46].

**Figure 13:**
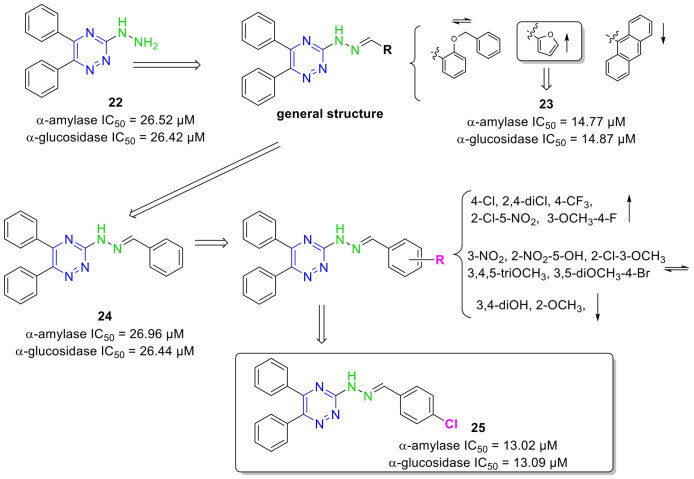
Structures of α-glucosidase inhibitors, as described by Shamin et al. [26].

Shamim et al. synthesised and evaluated **twenty-four** derivatives of 1,2,4-triazine (ten new compounds) as dual inhibitors of α-amylase and α-glucosidase [26]. All compounds showed inhibitory activity with IC_50_ values below 50 μM for both tested targets and were comparable to acarbose, the reference drug (α-amylase: IC_50_ = 12.94 ± 0.27 μM; α-glucosidase: IC_50_ = 10.95 ± 0.08 μM). These data were obtained in in vitro enzyme inhibition assays using α-amylase from *Aspergillus oryzae* and α-glucosidase from *Saccharomyces cerevisiae.* The authors also tested three intermediates from their syntheses, which themselves showed the ability to inhibit these enzymes (especially the hydrazine derivative **22**; Figure 13), indicating that the diphenyl-1,2,4-triazine moiety is mainly responsible for the observed biological activity.

Introducing a substituent into the hydrazine moiety had a varied effect on activity (Figure 13). The furan substituent (compound **23**; α-amylase: IC_50_ = 14.77 ± 0.02 μM; α-glucosidase: IC_50_ = 14.87 ± 0.04 μM) led to an increase in activity, whereas the phenyl substituent (compound **24**; α-amylase: IC_50_ = 26.96 ± 0.07 μM; α-glucosidase: IC_50_ = 26.44 ± 0.22 μM) had little effect on activity but provided an opportunity for further straightforward modifications. The introduced substituents in the benzene ring showed different effects on activity (Figure 13). Compound **25** (*para*-chloro-substituted) proved to be the most active among all tested compounds (α-amylase: IC_50_ = 13.02 ± 0.04 μM; α-glucosidase: IC_50_ = 13.09 ± 0.08 μM). SAR showed that the biological activity was closely related to the electronic nature and position of substituents on the aromatic ring. The presence of a strong electron-withdrawing substituent at the *para* position significantly improves ligand–enzyme interactions and overall inhibitory effectiveness. What is interesting is that all compounds showed similar activity for both targets. The values differed only in the tenths of a decimal point. To explain the inhibition mechanism for several of the most active compounds, including compounds **23** and **25**, kinetic studies were performed and analysed. Models, such as Lineweaver–Burk, Hill, Hanes–Woolf, Eadie–Hofstee, Dixon, and Scatchard, were used for this purpose. The plots obtained from the corresponding parameters indicated that all compounds acted as non-competitive inhibitors of α-amylase and as competitive inhibitors of α-glucosidase. Additionally, molecular docking studies were conducted on both biological targets to explain the mode of interaction between compounds and enzymes. The α-glucosidase homology model was built on *S. cerevisiae* (PDB: 3AJ7), whereas α-amylase was based on the crystallographic structure from PDB (PDB: 3BAJ). The results of these calculations showed good correlation with the experimental data.

#### 3.2.2. 1,2,4-Triazines as Multi-Target Ligands

**Figure 14:**
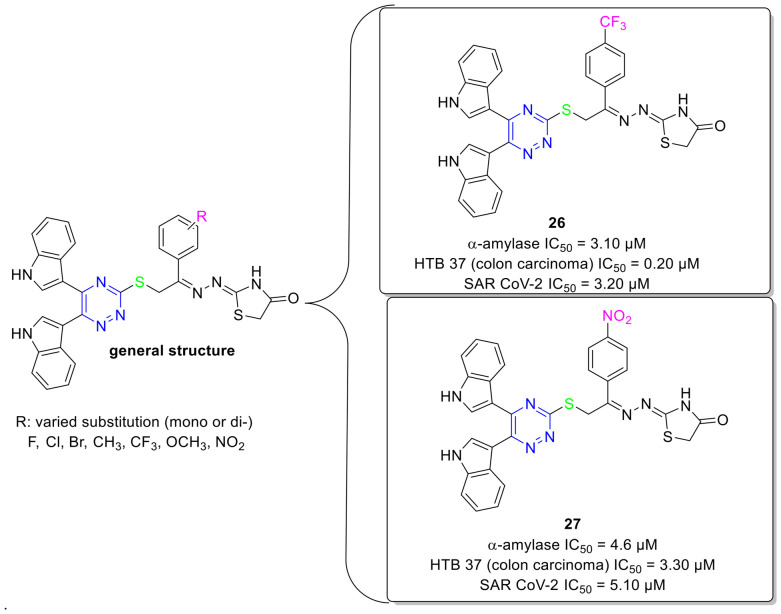
Structures of multi-target ligands, as described by Khan et al. [47].

Khan et al. published a series of **twelve** compounds designed as hybrids, combining bisindolo-1,2,4-triazine via a linker with a thiazolidine moiety to possess anticancer (colorectal cancer), antiviral (SARS-CoV-2), and antidiabetic (α-amylase inhibitors) activity [47]. All the synthesised derivatives exhibited moderate or strong inhibitory activity against the tested biological targets. Acarbose (α-amylase; IC_50_ = 7.30 ± 0.10 μM), tetrandrine (cancer cells; IC_50_ = 3.70 ± 0.20 μM), and GC-376 (IC_50_ = SARS-CoV-2) were used as standard drugs. SAR analysis showed that IC_50_ values were influenced by the substitution pattern in the benzene ring. Electron-withdrawing groups, such as CF_3_, F, and NO_2_, were important substituents for biological activity. Molecular docking studies, conducted for the most potent compounds against all biological targets, showed interactions responsible for inhibiting the enzymes. For α-amylase, there were hydrogen bonds between compounds and the residual amino acid enzymes. Among all compounds, compounds **26** and **27** were of particular interest (Figure 14).

## 4. Fused 1,2,4-Triazines as Antidiabetic Agents

### 4.1. 1,2,4-Triazolo-Fused 1,2,4-Triazines

#### 4.1.1. 1,2,4-Triazolo-1,2,4-triazines as Dual α-Amylase and α-Glucosidase Inhibitors

**Figure 15:**
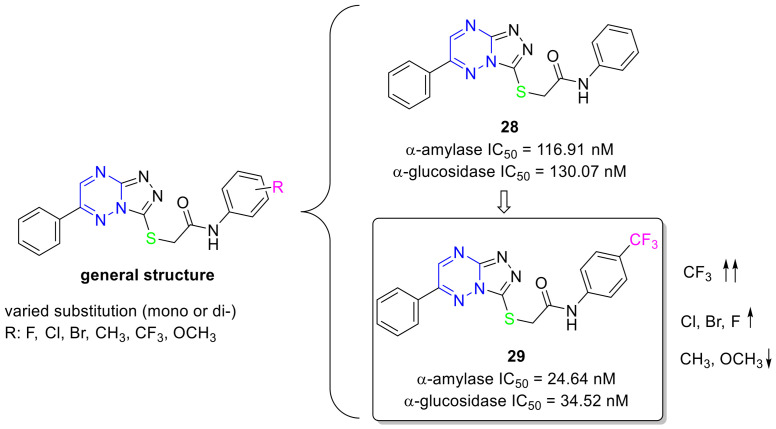
Structures of selected 1,2,4-triazolo-1,2,4-triazines as dual α-amylase and α-glucosidase inhibitors, as described by Seyfi et al. [48].

Based on the literature data, Seyfi et al. designed a series of 1,2,4-triazines as dual inhibitors of α-glucosidase and α-amylase [48]. These compounds were formed by joining two bioactive moieties: 1,2,4-triazine and 1,2,4-triazole. Both scaffolds are frequently found in molecules exhibiting a wide range of biological activities, including antidiabetic effects. **Fifteen** compounds, with the general structure shown in Figure 15, were tested for their inhibitory activity against α-amylase and α-glucosidase in vitro. All compounds exhibited inhibition of these enzymes in the nanomolar range (IC_50_: 24.64–115.57 nM for α-amylase and IC_50_: 34.52–213.44 nM for α-glucosidase). In most cases, the activity was superior or comparable (e.g., compound **28**; Figure 15) to that of acarbose (IC_50_: 112.47 nM for α-amylase and IC_50_: 151.73 nM for α-glucosidase). In general, the introduction of a substituent into the benzene ring increased activity against both enzymes (except that methoxy or methyl; Figure 15). The highest increase was observed for halogen substituents. The most potent compound in this series was compound **29** (Figure 15) with IC_50_ values of 24.64 nM for α-amylase and 31.87 nM for α-glucosidase. Further studies showed that this compound is a competitive inhibitor of α-glucosidase, with a K_i_ value of 33.85 ± 4.36 nM. Molecular docking to crystal structures of enzymes (PDB—no precise data) of compound **29** revealed interactions with specific proteins. In the case of α-amylase, the triazolo–triazine ring forms a π–π interaction with Trp59, whereas in the case of α-glucosidase, the benzene ring (attached to the triazolo–triazine core) forms a π–π interaction with Trp19 and nitrogen of the amide moiety with Asp225.

#### 4.1.2. 1,2,4-Triazolo-1,2,4-triazines as Dipeptyl Peptidase-4 Inhibitors

**Figure 16:**
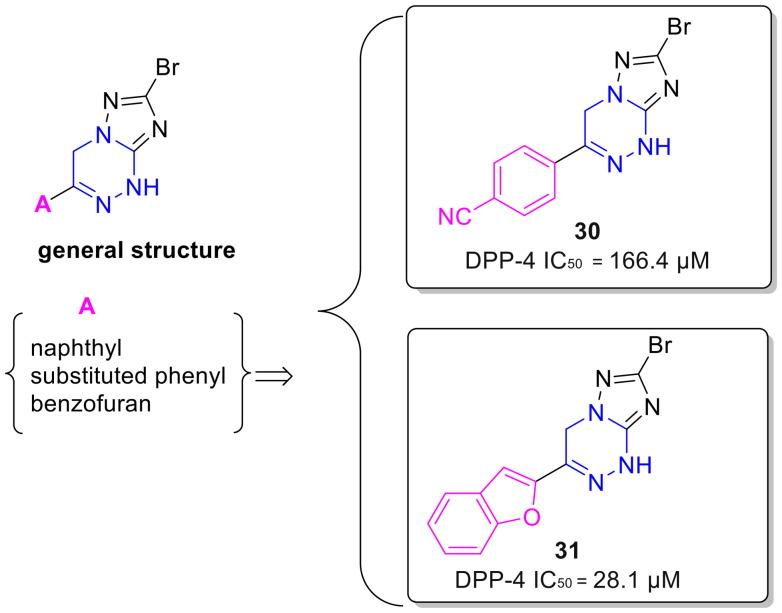
Structures and DPP-4 inhibitory activities of selected compounds, as described by Patel et al. [49].

Patel et al. described a search for new DPP-4 inhibitors among triazolo–triazines [49]. The compounds described in this study were selected for synthesis based on earlier in silico studies (3D-QSAR analysis, molecular docking, and virtual screening). **Seventeen** compounds were synthesised and subsequently tested for their ability to inhibit human DPP-4, as well as human DPP-8 and human DPP-9. DPP inhibitory activity was determined in the fluorescence-based enzyme assay. Of all the compounds, only two, compounds **30** and **31**, exhibited inhibitory activity for DPP-4, with IC_50_ values of 166.4 μM and 28.1 μM, respectively (Figure 16). Docking studies (PDB: 3KWF) showed that a benzofuran ring better fits the binding pocket than a phenyl ring. The most promising compound **31** was further investigated in in vivo studies. During oral glucose tolerance tests (in C57BL/6J mice), tested at three doses (5, 10 and 20 mg/kg), compound **31** reduced blood glucose levels in a dose-dependent manner. The highest result was observed for the dose of 20 mg/kg. Next, the antihyperglycaemic activity of this compound was evaluated in a chronic model of high-fat diet fed with streptozotocin in rats. This compound, tested at a dose of 14 mg/kg (daily oral) for 28 days, significantly reduced the glucose level only on the 28th day, whereas for sitagliptin (2 mg/kg; Figure 11), this effect was observed starting on the 14th day and was maintained throughout the testing period. Probably, this good in vivo effect of compound **31** is due to activity at targets other than DPP-4.

### 4.2. Pyrimido-1,2,4-Triazines

**Figure 17:**
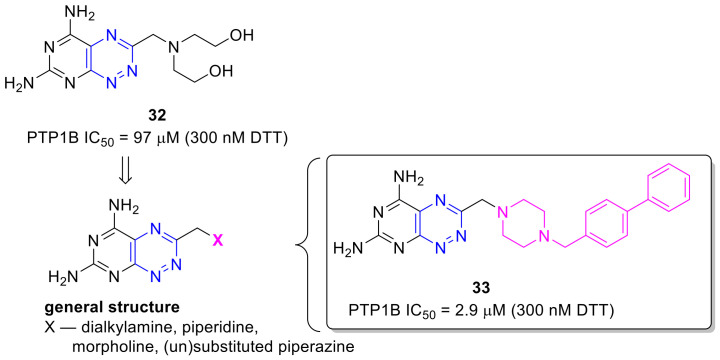
Structures and protein tyrosine phosphatase 1B inhibitory activities of selected compounds, as described by Guertin et al. [50].

Guertin et al. described pyrimido [5,4-e][1,2,4]triazine-5,7-diamines as hypoglycaemic agents [50]. The compounds inhibited protein tyrosine phosphatases (PTPs), including PTP1B. PTP1B acts as a key regulator of metabolism, particularly in the insulin and leptin signalling pathways, making this protein an ideal therapeutic target for the treatment of T2D and obesity. PTP1B negatively regulates insulin receptor signalling and contributes to insulin resistance [51]. The compounds were synthesised as structural modifications of compound **32** (Figure 17), which was identified through virtual screening. A series of **fifteen** compounds was tested for inhibitory activity against PTP1B using a fluorescence assay. IC_50_ values were measured in the presence of 300 nM and 2 mM dithiothreitol (DTT). All compounds (except one) showed inhibitory activity with IC_50_ values < 30 μM. These compounds inhibit PTP activity via a ‘vanadate-type’ redox mechanism, which involves the production of hydrogen peroxide or superoxide, which reversibly oxidise the cysteine residue responsible for the enzyme’s catalytic activity. The most active compound among these is shown in Figure 17. The glucose-lowering properties of compound **33** (Figure 17) were evaluated in vivo in male ob/ob mice. Compound **33** was tested at the dose of 50 mg/kg for 5 days. Furthermore, compound **33** showed a favourable half-life (t_1/2_ = 1 h) and excellent oral bioavailability (F = 97%). Its large volume of distribution (V_ss_ = 3.1 L/kg) indicated extensive tissue and cellular penetration.

### 4.3. 1,2,4-Triazinoindoles

#### 4.3.1. 1,2,4-Triazinoindoles as α-Glucosidase Inhibitors

**Figure 18:**
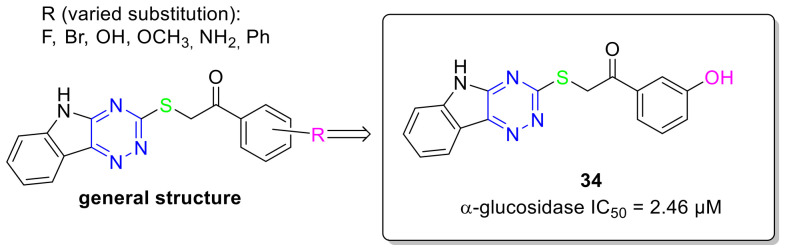
α-Glucosidase inhibitory activities of selected compounds, as described by Rahmin et al. [52].

Rahim et al. described a series of **eleven** 1,2,4-triazinoindole derivatives as α-glucosidase inhibitors [52]. α-Glucosidase inhibitory activity was evaluated spectrophotometrically by measuring the absorbance of *para*-nitrophenol formed at 400 nm from the *para*-nitrophenyl glycoside. Compounds showed inhibitory activity range from 2.26 to 312.79 μM. The most potent was a derivative with a 3-hydroxy substituent (compound **34**; Figure 18). The activity of some compounds was higher than that of the standard drug, acarbose (IC_50_: 38.25 ± 0.12 μM). The authors conducted molecular docking studies (PDB: 2AJ7) to explain this mode of action. Synthesised compounds showed interactions through the carbonyl oxygen and the 1,2,4-triazine moiety with the important active site residues. The highest activity (IC_50_ value 2.46 ± 0.01 μM) of compound **34** is connected with the presence of a hydroxyl substituent in the phenyl ring, and the oxygen and hydrogen of this group are both involved in hydrogen bonding with the most important residue, Asp349.

**Figure 19:**
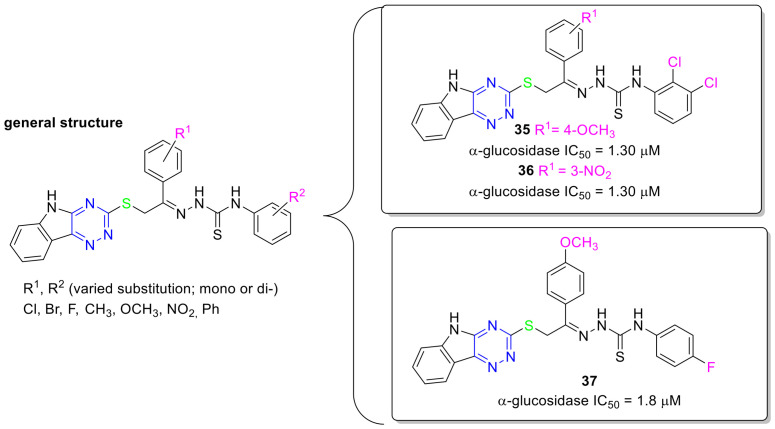
α-Glucosidase inhibitory activities of selected compounds, as described by Taha et al. [53].

Continuing this work, Taha et al. described a series of **twenty-five** triazinoindole derivatives as α-glucosidase inhibitors [53]. The compounds were designed based on earlier results published by Rahim et al. [52]. In this work, a thiosemicarbazide group was introduced into the compounds. The general structures of these is shown in Figure 19. All compounds were tested in the spectrofluorometric α-glucosidase assay. Acarbose, tested in this assay as a reference compound, had an IC_50_ value of 38.60 ± 0.20 μM. Compounds exhibited α-glucosidase inhibitory activity in the range of 1.3 to 35.80 μM. Seventeen compounds had IC_50_ values < 10 μM. The structures of the three most potent compounds (**35**–**37**) are shown in Figure 19. To explain their activity, these compounds were docked to the active sites of α-glucosidase from Sugar Beet (PDB code: 3W37). Before that, acarbose was docked to this enzyme. The results showed several interactions between structural elements of the molecules at these sites (especially hydrogen bonds within the cavity), which could be responsible for the observed inhibitory activity.

#### 4.3.2. 1,2,4-Triazinoindoles as α-Amylase Inhibitors

**Figure 20:**
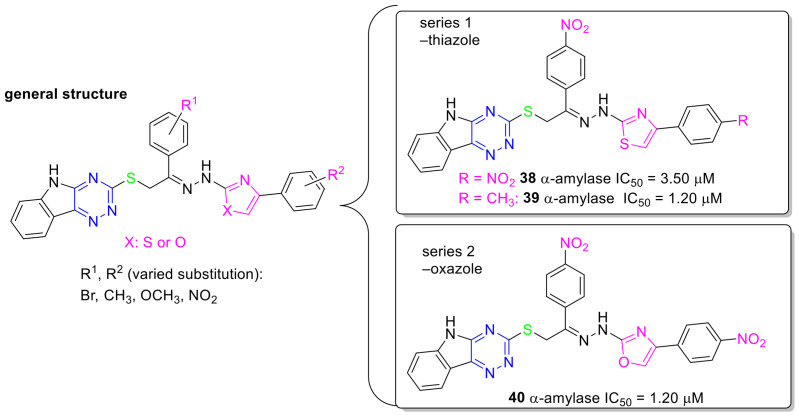
Selected compounds described by Rahim et al. as α-amylase inhibitors [54].

Continuing their work in the search for antidiabetic compounds among indolotriazines, Rahim et al. obtained a series of **twenty-one** compounds with the general structures shown in Figure 20 [54]. In this study, a thiazole or an oxazole ring was incorporated into the molecule. All compounds demonstrated the ability to inhibit α-amylase in the micromolar range (1.2–19.50 μM). In most cases this activity was weaker than that of acarbose (IC_50_ = 0.91 μM). SAR showed that the activity was influenced both by the type of heterocyclic ring introduced (thiazole, oxazole) and by the position and type of substituents on the benzene rings. The most active compounds **38**–**40** are shown in Figure 20. To confirm the biological inhibitory activity of compounds, Rahim et al. conducted molecular docking studies into the active site of the porcine α-amylase enzyme (PDB: 1OSE). The most potent compounds in both series showed favourable interactions with the active enzyme site. Compound **38** had good interactions with Arg195, Trp59, and H299, whereas compound **40** interacted with Tyr62, Thr163, Ap197, and Asp356. Generally, the docking results were well-correlated with the obtained compounds’ α-amylase inhibitory activities.

**Figure 21:**
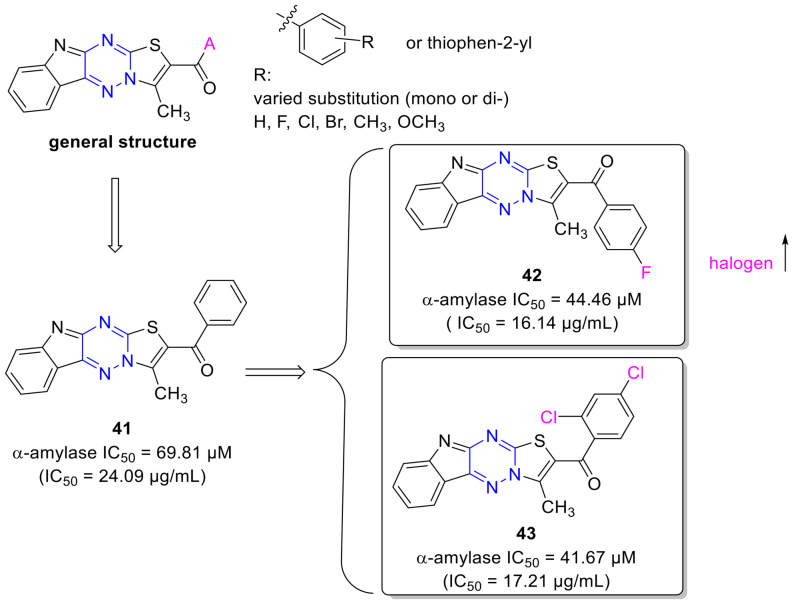
Selected compounds described by Aggarwal et al. as α-amylase inhibitors [55].

Aggarwal et al. described thiazolo-1,2,4-triazinoindoles as α-amylase inhibitors [55]. A series of **ten** compounds, with the general structures shown in Figure 21, was synthesised in solvent-free conditions. α-Amylase inhibitory activity was evaluated using α-amylase from *Aspergillus oryzae* and acarbose. Values of inhibitory activity were presented as IC_50_ but in μg/mL, so for better comparison with other results they were recalculated for μM [56]. All compounds showed lower inhibitory activity (IC_50_: 41.67–75.61 μM) than acarbose (IC_50_ = 28.87 ± 0.65 μM). Generally, the introduction of a substituent into the benzene ring increased inhibitory activity (comparing compound **41** vs. compounds **42** and **43**; Figure 21). The most potent compounds were **42** and **43**, with IC_50_ of 44.46 ± 1.13 μM and 41.67 ± 0.36 μM, respectively. Molecular docking studies of the tested compounds allowed evaluation of their binding mode within the active site of the α-amylase receptor (PDB code: 7TAA). Compounds **42** and **43** have similar binding properties to the reference ligand, acarbose.

#### 4.3.3. 1,2,4-Triazinoindoles as Aldose Reductase Inhibitors

**Figure 22:**
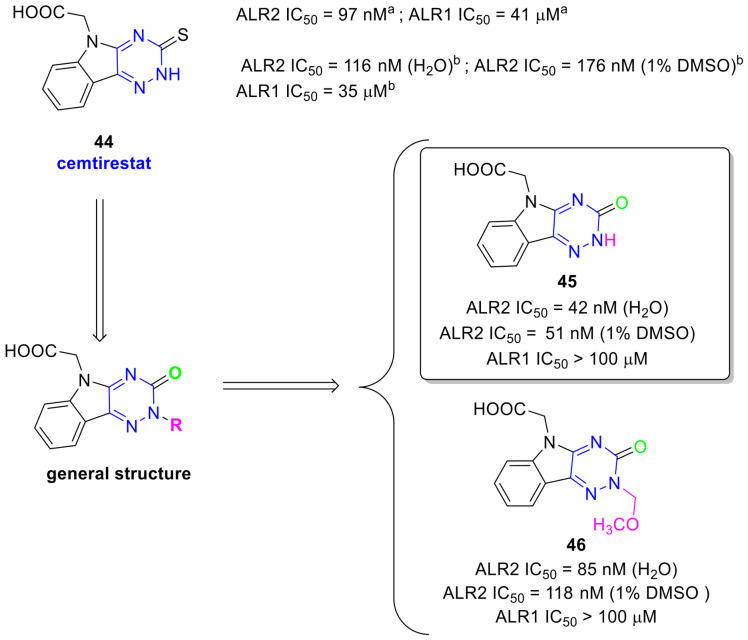
Structures of selected compounds as aldose reductase inhibitors, as described by Hlavac et al. [62]. ^a^ data from Ref. [57]; ^b^ data from Ref. [62].

In 2015, Stefek et al. described results from a ligand-based search of the ChemSpider database to find compounds containing an indole-1-acetic acid and indoline-1-acetic acid scaffold as aldose reductase inhibitors (ARIs) [57]. From the initial 5813 compounds after further requirements, **fifteen** compounds were selected for in vitro testing. Of these, compound **44** (Figure 22) showed the strongest and most selective activity, with an IC_50_ for ALR2 of 97 nM and for ALR1 of 40.6 μM. Further testing of this compound showed an uncompetitive inhibition of ALR2 with a K_i_ of 0.089 μM/L. In additional studies, the inhibitory effect of compound **44** was confirmed in isolated rat eye lenses exposed to high glucose (50 mM; 4h incubation). Dose-dependent sorbitol accumulation was observed from the concentration of 10 μM. Crystal structure of ALR2 with compound **44** explained high activity of this compound. Furthermore calculated ADMET parameters showed that this compound was a promising candidate for further ARI development. Subsequent studies confirmed the safety of this compound (called cemtirestat), its strong antioxidant and ROS-modulating effects, its anti-inflammatory signalling suppression, and its neuroprotection [58,59,60,61]. Continuing the work in this field, Hlaváč et al. developed a series of **four** oxotriazinoindoles based on the structure of cemtirestat (Figure 22) [62]. The compounds were evaluated as inhibitors of ALR2 using an in vitro aldose reductase inhibition assay based on the reduction in *D,L-glyceraldehyde* catalysed by ALR2, isolated from a rat lenses with NADPH as a cofactor.

Activity was evaluated in water and in water with 1% DMSO. IC_50_ values for ALR2 are in the nanomolar range (IC_50_ < 800 nM). Generally, compounds showed lower (1.2–1.9 times) activity in 1% DMSO. Selectivity for ALR1, isolated from rat kidney using *D-glucuronate* as the substrate, was also checked. All compounds (except one) had inhibitory activity higher than 100 μM [55]. Structures of the strongest inhibitors, compounds **45** and **46**, are shown in Figure 22.

Compound **45** exhibited the highest inhibitory potency toward ALR2 with an IC_50_ value of 42 ± 1 nM (in water) and 51 ± 1 nM (in 1% DMSO). For ALR1, the IC_50_ value exceeded 100 μM, with 24% inhibition at 100 μM, indicating very high selectivity for ALR2 with a selectivity factor > 2380. For comparison, the reference inhibitor cemtirestat showed ALR2 IC_50_ values of 116 ± 8 nM (water) and 176 ± 1 nM (1% DMSO), with ALR1 IC_50_ of 35 ± 2 μM. These results demonstrate that replacement of sulphur with oxygen in the heterocyclic core significantly enhances ALR2 inhibition while maintaining minimal activity toward ALR1. Moreover, compound **45** inhibited human recombinant AKR1B1 with an IC_50_ value similar to that of the rat ALR2 (IC_50_ = 66 nM) but with much weaker strength against human AKR1B10 (IC_50_ = 56,240 nM). Furthermore, compound **45** did not show significant inhibitory effect on SDH activity, even at concentrations up to 100 μM. Ex vivo studies performed on isolated rat eye lenses demonstrated that compound **45** markedly decreased sorbitol accumulation at concentrations as low as 10 μM, with inhibition reaching nearly 75% at 50 μM.

## 5. 1,3,5-Triazines as Antidiabetic Agents

### 5.1. Imeglimin

**Figure 23:**
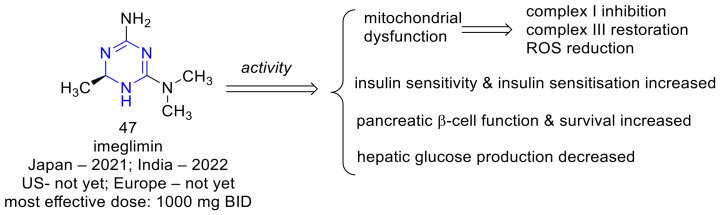
Chemical structure of imeglimin and its pharmacological activity. BID, twice daily.

**Imeglimin** (compound **47**; Figure 23), a new antidiabetic drug for the treatment of T2D, contains a tetrahydrotriazine moiety and represents the first *glimins* drug [63]. Its mechanism of action differs from traditional antidiabetic drugs because it increases mitochondrial function, which plays a crucial role in the pathogenesis of insulin resistance. Moreover, imeglimin enhances β-cell insulin secretion and reduces hepatic gluconeogenesis. The biological activity of imeglimin was evaluated in several preclinical and clinical studies. A few systematic reviews and meta-analyses summarise properties, mechanisms of action, and efficacy and safety in preclinical and clinical trials [64,65,66,67,68]. In preclinical experiments with Zucker diabetic fatty (ZDF) rats, animals were treated with imeglimin for five weeks at a dose of 150 mg/kg twice daily. The results demonstrated a significant improvement in glucose-stimulated insulin secretion (GSIS) in the insulinogenic index during glucose tolerance testing. Additionally, imeglimin increased pancreatic β-cell mass, reduced β-cells apoptosis, and stimulated cell proliferation, indicating a protective effect on pancreatic islets. Further studies revealed that imeglimin acts at multiple metabolic sites, including the pancreas, liver, and skeletal muscle. At the mitochondrial level, the compound enhances ATP production, increases NAD^+^ synthesis by activation of the salvage pathway, and reduces excessive ROS formation. These effects improve cellular energy metabolism and prevent mitochondrial stress-induced cell death. Moreover, imeglimin suppresses hepatic gluconeogenesis and enhances peripheral glucose uptake in muscle cells via an AMPK-dependent pathway, thereby improving insulin sensitivity. The clinical efficacy and safety of imeglimin were further evaluated in the TIMES clinical trial programme conducted in Japan.

In the TIMES 1 phase III trial, imeglimin monotherapy (1000 mg twice daily) was compared with placebo in 213 patients with T2D during a 24-week study period. The results demonstrated a significant reduction in glycated haemoglobin (HbA1c decreased by 0.87%) with a safety profile comparable to placebo. In the TIMES 2 trial, which included 714 patients, imeglimin was evaluated both as monotherapy and in combination with other antidiabetic drugs. After 52 weeks of treatment, imeglimin significantly reduced HbA1c levels by 0.46–0.92%, depending on the concomitant therapy. The strongest reduction was observed when imeglimin was combined with DPP-4 inhibitors, whereas a weaker effect was noted in combination with GLP-1 receptor agonists. Importantly, the drug demonstrated a favourable safety profile, with mostly mild adverse events and a relatively low risk of hypoglycaemia. The TIMES 3 study further investigated imeglimin in combination with insulin therapy in 215 patients with T2D. After 16 weeks of treatment, HbA1c levels decreased by 0.60%, and this reduction was maintained during a 52-week extension period. The study confirmed that imeglimin can be safely combined with insulin without significantly increasing the risk of hypoglycaemia.

Additional clinical evidence was obtained from studies with continuous glucose monitoring systems. In this study involving 32 patients, administration of imeglimin significantly improved glycaemic parameters, reducing mean glucose levels from 159.0 ± 27.5 mg/dL to 141.7 ± 22.1 mg/dL, while increasing time-in-range values and decreasing time above range.

To summarise, imeglimin is well-tolerated, although gastrointestinal symptoms (nausea, diarrhoea) occur more frequently at doses above 2000 mg/day, and can be used both as monotherapy and in combination with other drugs (metformin, DPP-4 inhibitors, insulin).

Positive results from clinical trials led to the drug’s approval in Japan in 2021 and in India a year later. To date, the drug has not been approved in the United States or Europe [63].

### 5.2. Arylated Imeglimin Derivatives as Potential Antidiabetic Agents

**Figure 24:**
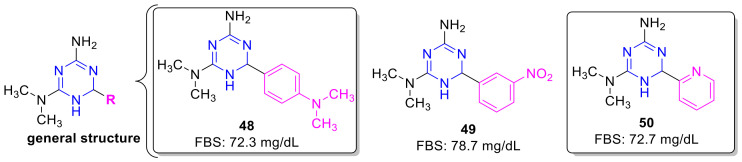
Structures of the most active imeglimin derivatives, as described by Khodakhah et al. [69]. FBS, fasting blood sugar.

Khodakhah et al. reported the development and biological evaluation of a series of imeglimin-derived compounds containing a 1,3,5-triazine scaffold as potential antidiabetic agents. The authors synthesised **ten** aryl-substituted derivatives, such as compounds **48**–**50** (Figure 24), by condensation of metformin with various aromatic aldehydes under reflux conditions in acetic acid [69].

The biological activity of compounds **48**–**50** was evaluated in vivo using a zebrafish (*Danio rerio*) diabetic model, which is commonly applied in metabolic studies due to its physiological similarities to mammalian glucose regulation. Hyperglycaemia was induced by exposing zebrafish to gradually increasing glucose concentrations, resulting in significantly elevated fasting blood sugar (FBS) levels. The animals were divided into experimental groups and treated with the synthesised compounds at a concentration of 10 μM for 48 h, while metformin and imeglimin were used as reference drugs (10 μM). After treatment, FBS levels were determined using a glucometer following blood collection from the caudal fin under anaesthesia.

The obtained results demonstrated that all tested derivatives reduced blood glucose levels in the diabetic zebrafish model. The untreated diabetic group showed a markedly elevated FBS level of 153.3 ± 15.5 mg/dL, whereas the non-diabetic control group exhibited physiological values of 75.7 ± 4.4 mg/dL. Compounds **48**–**50** showed the most pronounced antihyperglycaemic effects. In particular, compounds **48** and **50** demonstrated the highest activity, reducing FBS levels to 72.3 ± 7.2 mg/dL and 72.7 ± 4.3 mg/dL, respectively, which was comparable to the effect observed for metformin (74.0 ± 5.1 mg/dL) and more effective than imeglimin (82.3 ± 5.2 mg/dL).

To further rationalise the experimental results, molecular docking studies were performed for the most active compounds **48**–**50** against two proteins involved in glucose metabolism, sirtuin 1 (SIRT1) (PDB: 5BTR) and glycogen synthase kinase-3β (GSK-3β) (PDB: 1Q4L). The docking simulations indicated favourable binding affinities for all three compounds, with compound **48** exhibiting the strongest predicted interactions with both targets. The calculated binding energies were significantly lower than those for metformin, suggesting stronger interactions with the enzyme’s active sites. The predicted binding modes involved electrostatic and π–alkyl interactions with key amino acid residues located within the catalytic pocket. These computational findings were consistent with the in vivo biological data and supported the hypothesis that modulation of SIRT1 and GSK-3β activity may contribute to the observed antihyperglycaemic effects of the investigated compounds.

### 5.3. 1,3,5-Triazines with Thiazolidinedione Moiety as Antidiabetic Agents

**Figure 25:**
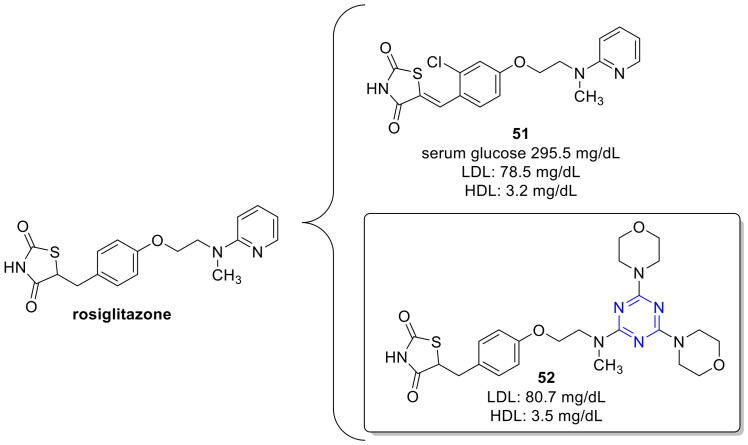
Thiazolidinedione drugs and structures of compounds, as described by Ahmadi et al. [71]. LDL, low-density lipoprotein; HDL, high-density lipoprotein.

Thiazolidinediones (TZDs) constitute an important class of antidiabetic agents that act mainly as insulin sensitisers by activating the peroxisome proliferator-activated receptor gamma (PPARγ). A few representatives of this group have entered the market, but only one is now in use, i.e., pioglitazone (Figure 22). The others were withdrawn due to hepatotoxicity (e.g., ciglitazone) or heart failure (e.g., rosiglitazone). TZDs share a common pharmacophore containing a polar thiazolidinedione ring, an aromatic phenyl linker, and a hydrophobic tail fragment [70]. Ahmadi et al. reported the synthesis and biological evaluation of **two** 2,4-thiazolidinediones (compounds **51** and **52**; Figure 25) designed as structural modifications of rosiglitazone [71]. In compound **52**, the lipophilic pyridyl ring of rosiglitazone was replaced with a hydrophilic dimorpholino–triazine moiety. The antihyperglycaemic and hypolipidaemic activities of the compounds were evaluated in vivo in alloxan-induced diabetic rats (alloxan: 150 mg/kg i.p.). Both compounds exhibited antihyperglycaemic activity comparable to rosiglitazone and improved lipid parameters. Compound **52** significantly decreased LDL levels with 80.7 ± 7.2 mg/dL and increased HDL levels with 3.5 ± 0.7 mg/dL. The effect was higher than observed for rosiglitazone (90.6 ± 8.6 mg/dL; 1.7 ± 0.6 mg/dL). The results suggest that structural modification of the TZD scaffold may enhance both antihyperglycaemic and antihyperlipidaemic activity [71].

### 5.4. 1,3,5-Triazines as DPP-4 Inhibitors

Andrews et al. [72] described a way to identify a potent, selective, and orally bioavailable drug candidate to replace existing therapies with DPP-4 inhibitors.

**Figure 26:**
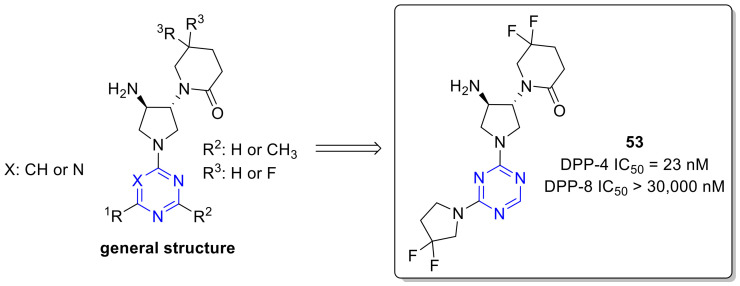
DPP-4 inhibitors, as described by Andrews et al. [72].

Based on earlier efforts from Pfizer laboratories to find DPP-4 inhibitors, they obtained compounds with a 3-aminopyrrolidine moiety connected to pyrimidine or 1,3,5-triazine ring. Among all **sixteen** compounds, three contained a 1,3,5-triazine moiety and showed high inhibitory activity against the DPP-4 enzyme (IC_50_ < 60 nM). Of these, compound **53** (Figure 26) exhibited an IC_50_ of 23 nM for DPP-4 and high selectivity vs. DPP-8 (IC_50_ > 30 μM).

This compound was further tested in many in vitro assays to evaluate its pharmacological and ADME properties. Results showed a K_i_ of 14.4 nM for DPP-4 in human plasma and >1000-fold selectivity over related proteases, including DPP-2, DPP-3, and DPP-9. Other studies revealed good selectivity (CEREP evaluation in 68 assays IC_50_ > 10 μM, CYP1A2, CYP2C9, CYP2D6, CYP3A4) and acceptable PK properties. Thus, compound **53** represented a good starting structure for the search for new DPP-4 inhibitors.

**Figure 27:**
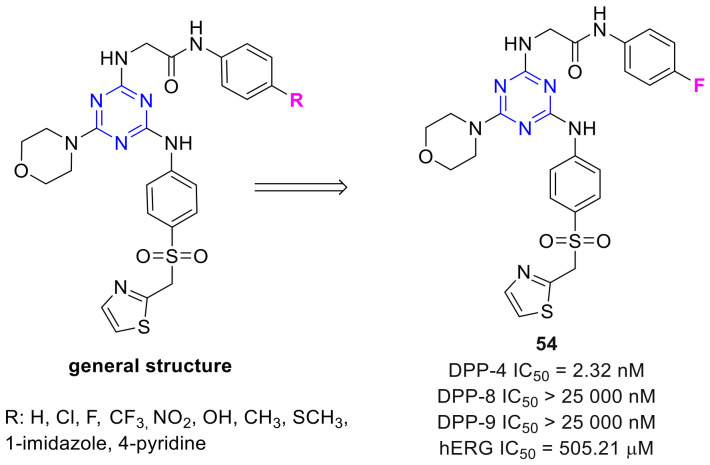
DPP-4 inhibitors described by Gao et al. [73].

Gao et al. [73] described studies on DPP-4 inhibitors among 1,3,5-triazine-thiazole sulphonamide derivatives. These compounds were designed as hybrids combining moieties found in compounds exhibiting antidiabetic activity, i.e., the thiazole, triazine, and sulphonamide groups. **Ten** compounds were obtained and tested not only for their ability to inhibit DPP-4 but also for their ability to inhibit DPP-8 and DPP-9. All compounds exhibited DPP-4 inhibition with IC_50_s values below 600 nM. The general structures and the best compound, compound **54**, are shown in Figure 27. SAR analysis revealed that the introduction of a substituent into the benzene ring resulted in increased activity. Electron-withdrawing substituents proved to be more favourable than electron-donating substituents. Furthermore, the effect of the compounds on hERG channels was investigated. The compounds exhibited significantly weaker activity than at DPP-4. Additionally, docking to DPP-4 (PDB: 2FJP) was performed for compound **54**. This compound successfully docked to all three specific regions of the enzyme, namely S1, the N-terminal region, and S2. Compound **54** was also tested in vivo assays: in an oral glucose tolerance test (male IRC mice) and in STZ-induced diabetes in rats. Compound **54** was tested at three doses (3 mg/kg, 10 mg/kg, and 30 mg/kg). The strongest effects were observed in the groups administered 30 mg/kg. Additionally, the effect of compound **54** on antioxidant enzymes in normal and diabetic rats was assessed. An improvement in the levels of some enzymes was observed; for example, in superoxide dismutase (SOD), catalase (CAT), and glutathione peroxidase (GPx).

### 5.5. 1,3,5-Triazines as Glycolytic Enzyme Enolase Inhibitors

**Figure 28:**
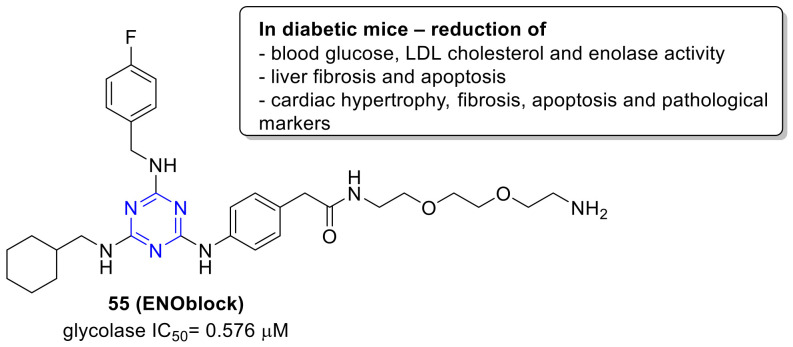
Structure of **ENOblock** and pharmacological activity in diabetic mice, as described by Cho et al. [74].

Cho et al. investigated a novel small-molecule inhibitor of the glycolytic enzyme enolase named **ENOblock** (**55**; Figure 28), which modulates the non-glycolytic “moonlighting” functions of this enzyme [74]. This compound was found by Jung et al. [75] during a screening study of small molecules in a cancer cell assay. Investigation of ENOblock showed unrecognised roles of enolase in cancer progression and gluconeogenesis. In zebrafish studies, inhibition of enolase by ENOblock reduced metastasis of cancer cells in vivo. Furthermore, it inhibited the action of a gluconeogenesis regulator PEPCK, thus representing a new target in the development of antidiabetic drugs. The work described by Cho et al. [74] is a continuation of the biological evaluation of ENOblock. Initial in vitro experiments were performed in insulin-responsive cell lines, including 3T3-L1 preadipocytes and Huh7 hepatocytes. Treatment with ENOblock (10 µM) significantly decreased enolase enzymatic activity and induced nuclear translocation of the enzyme, where it acts as a transcriptional repressor. This effect resulted in downregulation of known enolase target genes, such as *c-Myc* and *Erbb2*, demonstrating that ENOblock modulates gene expression by regulating enolase localisation. The antidiabetic potential of ENOblock was further evaluated in vivo using the db/db mouse model of T2D. Mice were treated with ENOblock at doses of 8 mg/kg or 12 mg/kg for seven weeks, and the results were compared with those obtained for rosiglitazone (8 mg/kg). ENOblock treatment significantly reduced blood glucose levels and decreased serum LDL cholesterol and free fatty acid concentrations. In addition, the compound reduced enolase activity in liver and kidney tissues and suppressed the expression of genes involved in gluconeogenesis and lipid metabolism, including *Pck-1* and *Srebp-1*. Further histological and molecular analyses revealed that ENOblock exerted additional beneficial effects in diabetic mice. The compound reduced liver fibrosis, inflammation, and apoptosis, as evidenced by decreased expression of inflammatory markers, such as *TNF-α* and *IL-6*. Similar protective effects were observed in adipose tissue, the heart and the kidney, where ENOblock treatment decreased tissue fibrosis, reduced apoptosis, and improved metabolic parameters. Importantly, compared with rosiglitazone, ENOblock produced less liver lipid accumulation and fewer pathological changes in cardiac tissue, indicating a potentially improved safety profile [74]. This compound is commercially available (also under the name A-III-a4) as a non-substrate analogue of an enolase inhibitor, which can be used in research in cancer and diabetes.

### 5.6. 1,3,5-Triazine as Sorbitol Dehydrogenase Inhibitors

**Figure 29:**
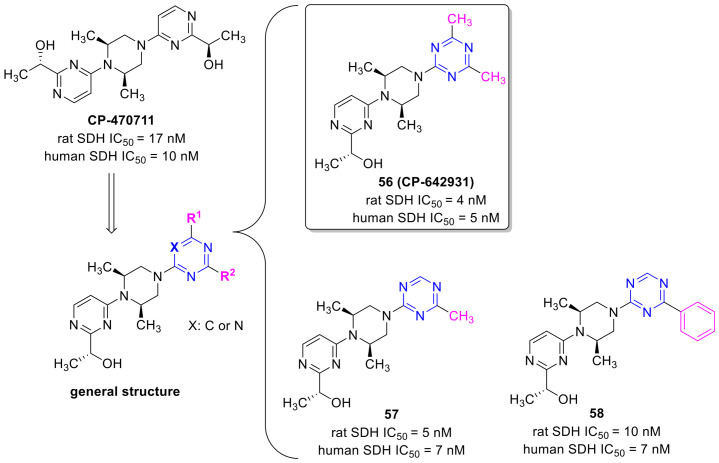
Structures of sorbitol dehydrogenase inhibitor CP-470711 and selected compounds, as described by Mylari et al. [76].

Sorbitol dehydrogenase (SDH) is an enzyme in the polyol pathway that converts sorbitol to fructose using NAD+. Under hyperglycaemic conditions, increased activity of this pathway disrupts the NAD+/NADH balance, causing oxidative stress and contributing to diabetic complications, such as neuropathy, retinopathy, and nephropathy. Studies using inhibitors of these enzymes have shown inconsistent results. In several studies, SDH inhibition worsened the course of neuropathy, increased oxidative stress, or accelerated tissue damage. In contrast, other studies have shown that SDH inhibition improved early vascular abnormalities (e.g., retinal vascular permeability) [76]. Mylari et al. described the design and biological evaluation of **ten** compounds as SDH inhibitors (SDIs) [77]. The compounds were based on the structure of the SDI inhibitor CP-470711 (Figure 29) described by Chu-Moyer [78] and contained a pyrimidine core connected to a 1,3,5-triazine moiety via a dimethylpiperazine linker (general structure; Figure 29). The pharmacological activity of compounds was evaluated in both in vitro and in vivo studies. Enzymatic assays were performed on recombinant rat and human SDH. In vivo activity was evaluated in the streptozotocin-induced diabetic rats (in acute and chronic models) by measuring the compounds’ ability to reduce elevated fructose levels in the sciatic nerve. This parameter reflects inhibition of SDH activity within the polyol pathway. All investigated derivatives exhibited good inhibitory activity, with IC_50_ values < 50 nM. Structures of the most promising compounds (**56**–**58**) are shown in Figure 29.

Compound **56** inhibited rat SDH, with an IC_50_ value of approximately 4 nM, and human SDH, with an IC_50_ of about 5 nM. In the chronic diabetic rat model, this compound demonstrated very strong in vivo activity, with an ED_90_ value of 0.05 mg/kg, indicating highly efficient inhibition of fructose accumulation in the sciatic nerve. Compound **57** also exhibited strong inhibitory activity, with IC_50_ values of 5 nM against rat SDH and 7 nM against human SDH, as well as an ED_90_ value of 0.3 mg/kg in the chronic model. Slightly lower potency was observed for compound **58**, which inhibited rat and human SDH, with IC_50_ values of 10 and 7 nM, respectively, and showed an ED_90_ value of 1 mg/kg.

Compound **56** was further evaluated in pharmacokinetic studies and exhibited good solubility in simulated gastric fluid (>1.3 mg/mL), moderate lipophilicity (log P ≈ 2.0), and efficient permeability across Caco-2 cell monolayers (Papp > 10^−5^ cm/s). Additionally, the serum half-life of compound **56** was approximately 7 h in rats and 10 h in dogs, supporting sustained biological activity. Consistent with these properties, administration of compound **56** produced prolonged inhibition of fructose accumulation in the sciatic nerve of diabetic rats for more than 24 h. Overall, the results demonstrated that optimisation of the triazine–pyrimidine scaffold led to highly potent SDI, compound **56**, which is commercially available for diabetic and cardiovascular studies (CP-642931). Compound **56** reached clinical trials, but when tested in healthy participant (1–35 mg daily for 7 days), it was not well-tolerated due to adverse neuromuscular effects [79].

**Figure 30:**
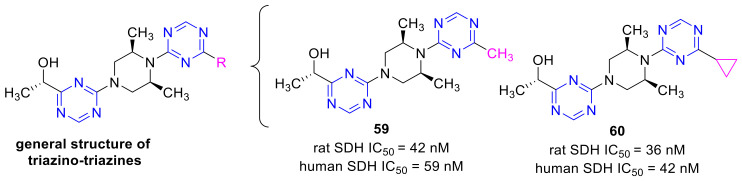
Structures of selected sorbitol dehydrogenase inhibitors with triazino–triazine cores, as described by Mylari et al. [80].

Continuing the work in this field, Mylari et al. described, in a subsequent study in 2003 [80], where two series with (R)-hydroxyethylpyridine and (R)-hydroxyethyltriazine cores were evaluated. In triazino–triazine series (general structure in Figure 30), **five** compounds were described. They showed SDH inhibitory activity with IC_50_ values < 500 nM. The most potent SDIs proved to be compounds **59** and **60** (Figure 30).

Compound **59** exhibited high potency with IC_50_ values of approximately 42 nM (rat SDH) and 59 nM (human SDH). In vivo, it achieved near-complete normalisation of sciatic nerve fructose levels, reaching 114% in the acute model and 96% in the chronic model at a dose of 10 mg/kg. Similarly, compound **60** displayed high in vitro potency (IC_50_ ≈ 36–42 nM) and strong in vivo activity, with approximately 87% and 77% normalisation of fructose levels in acute and chronic models, respectively, at doses of 3–9 mg/kg. The enantiomer of compound **60** was the least potent inhibitor in the whole series, with an IC_50_ of 400 nM for rat SDH and 390 nM for human SDH.

Overall, both studies showed that compounds with triazino–triazine moiety (compounds **59**, **60**) are less potent than compounds with triazino–pyrimidine group (compounds **56**–**58**). Furthermore, SDI activity depended on a combination of factors, including strong zinc-binding capability, optimal lipophilicity, and stereochemical integrity. Importantly, the use of both in vitro and in vivo studies provided a comprehensive evaluation of these compounds, enabling correlation between enzyme-inhibition potency and therapeutic efficacy.

### 5.7. 1,3,5-Triazines as Potential Agents for Diabetic Nephropathy

**Figure 31:**
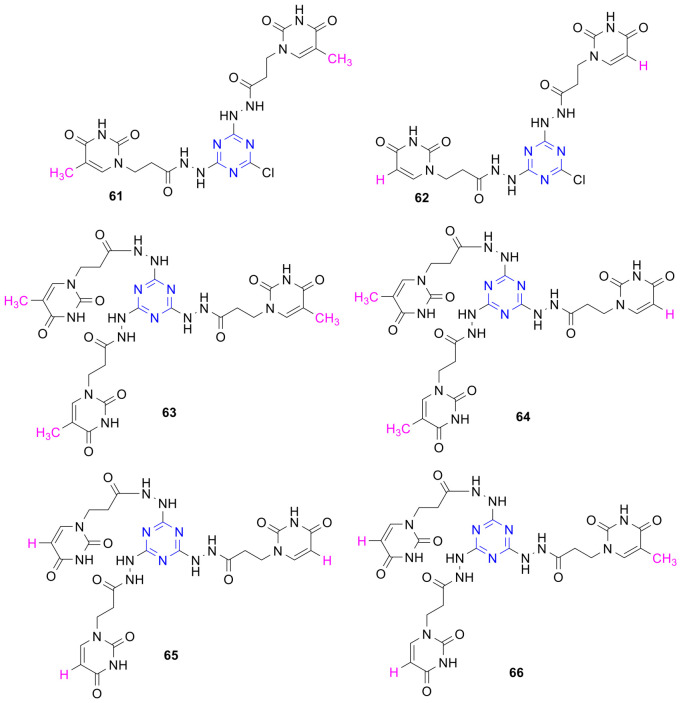
Representative structures of di- and trisubstituted 1,3,5-triazines incorporating uracil or thymine fragments, as described by El-Harakeh et al. [81].

El-Harakeh et al. [81] described the design and biological evaluation of **eight** novel triazine-based pyrimidine derivatives as potential therapeutic agents for diabetic nephropathy. The authors synthesised a series of mono-, di-, and trisubstituted 1,3,5-triazines containing nucleobase fragments, particularly uracil and thymine. Among the prepared compounds were **61**–**66**, which differ in the degree of substitution of the triazine ring and the nature of the nucleobase substituent (Figure 31).

The biological activity of the synthesised derivatives was evaluated in vitro using cultured rat glomerular mesangial cells exposed to high glucose conditions that mimic the diabetic environment. Cells were incubated for 48 h with high glucose (HG, 25 mM), and the expression of fibronectin, an important extracellular matrix protein associated with the progression of diabetic nephropathy, was determined by Western blot analysis. Normal glucose conditions (NG) were used as the control. Exposure to high glucose significantly increased fibronectin expression compared with the control level (NG = 100%), reaching approximately 270% of the control value under hyperglycaemic conditions. Among the investigated molecules, the trisubstituted triazines **61**–**64** (Figure 31) exhibited the strongest biological activity. At a concentration of 0.5 μM these compounds significantly reduced HG-induced fibronectin expression, with **64** showing the most pronounced inhibitory effect. In contrast, the disubstituted derivatives **61** and **62** (Figure 31) demonstrated weaker activity, indicating that the degree of substitution on the triazine core plays an important role in determining the biological response. The reported results were statistically significant compared with HG conditions (*p* < 0.05). Since mesangial cell proliferation represents an important pathological process during the early stages of diabetic nephropathy, the influence of the most active compounds **63**–**66** on cell growth was further investigated using the MTT assay. Rat glomerular mesangial cells were treated with HG (25 mM) for 48 h in the presence or absence of the tested derivatives at concentrations of 0.5 and 1 μM. The results demonstrated that hyperglycaemic conditions significantly increased mesangial cell proliferation, whereas treatment with **63**–**66** markedly attenuated HG-induced cellular growth. The antioxidative properties of the active derivatives were also investigated by measuring ROS production. ROS levels were determined using dihydroethidium fluorescence combined with HPLC analysis. High glucose markedly increased ROS generation in mesangial cells, whereas the presence of compounds **63**–**66** significantly reduced ROS levels, particularly at concentrations between 0.5 and 1 μM. Furthermore, these derivatives inhibited NADPH-oxidase-dependent superoxide generation, which represents one of the major sources of oxidative stress involved in the development of diabetic nephropathy [81].

Overall, the results indicate that trisubstituted triazines **61**–**66** effectively reduce extracellular matrix protein accumulation, inhibit mesangial cell proliferation, and suppress oxidative stress under hyperglycaemic conditions. These biological effects are highly relevant for the treatment of diabetic nephropathy, as oxidative stress and mesangial expansion are key pathological mechanisms responsible for the progression of diabetic kidney damage. Therefore, these compounds may represent promising lead structures for the development of new therapeutic agents targeting diabetic nephropathy [81].

### 5.8. Triazine-Based Insulin Mimetics Identified by Fluorescent Glucose Uptake Screening

Jung et al. reported the identification of **six** novel triazine-based insulin mimetics as potential antidiabetic agents for the treatment of diabetes mellitus, particularly in cases associated with impaired insulin secretion or insulin resistance [82]. Since insulin is the primary hormone responsible for regulating glucose homeostasis, compounds capable of mimicking insulin action may provide an alternative therapeutic strategy by enhancing peripheral glucose uptake and reducing hyperglycaemia. In this study, the authors developed a fluorescence-based screening platform utilising 6-NBDG (6-(*N*-(7-nitrobenz-2-oxa-1,3-diazol-4-yl)amino)-6-deoxyglucose), a non-metabolizable fluorescent glucose analogue, to identify small molecules capable of stimulating cellular glucose uptake. This assay was designed as a rapid and cost-effective alternative to conventional radioactive glucose uptake methods and enabled screening of a chemical library comprising 576 triazine-based small molecules in differentiated 3T3-L1 adipocytes, which were selected due to their high insulin sensitivity and strong GLUT4-mediated glucose transport response.

**Figure 32:**
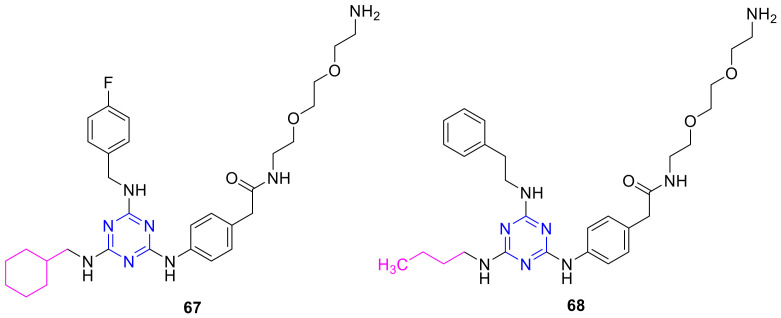
Representative triazine-based insulin mimetic compounds as novel glucose uptake stimulators with antidiabetic potential [82].

Following primary screening, **four** compounds were identified as enhancers of glucose uptake. However, additional apoptosis and cytotoxicity assays demonstrated that two candidates induced cell death and were therefore excluded from further investigation. The remaining non-toxic lead compounds, **67** and **68** (Figure 32), were selected as the most promising representatives of the investigated series. Their insulin mimetic properties were further validated using GLUT4 inhibition assays with cytochalasin B and 4,6-ethylidine-D-glucose, confirming that the observed increase in glucose uptake occurred through glucose transporter-mediated pathways. To further establish their biological relevance, the compounds were subjected to free fatty acid release assays, a classical method used to confirm insulin mimetic behaviour, where both derivatives successfully inhibited epinephrine-stimulated lipolysis similarly to known insulin mimetic controls. Additional testing in TNF-α-induced insulin-resistant adipocytes demonstrated reduced activity under insulin-resistant conditions, further supporting a mechanism of action closely related to insulin signalling pathways. Importantly, both lead compounds exhibited biological activity at concentrations of 5 μM, whereas the reference insulin mimetic zinc sulfate required 250 μM to achieve comparable effects, indicating substantially greater potency of the triazines. Beyond their glucose-lowering potential, compounds **67** and **68** also demonstrated beneficial secondary anti-inflammatory effects, as shown by their ability to reduce monocyte adhesion to endothelial cells and suppress VCAM-1 expression under hyperglycaemic conditions, suggesting possible protective effects against diabetes-associated vascular complications such as atherosclerosis. Collectively, the results indicate that compounds **67** and **68** constitute a promising new subclass of triazine-based insulin mimetic compounds with dual antidiabetic and vasculoprotective properties, highlighting the therapeutic potential of triazine scaffolds for future antidiabetic drug development.

### 5.9. 1,3,5-Triazines as Multi-Target Antidiabetic Agents

#### 5.9.1. 1,3,5-Triazines with Antibacterial Activity

**Figure 33:**
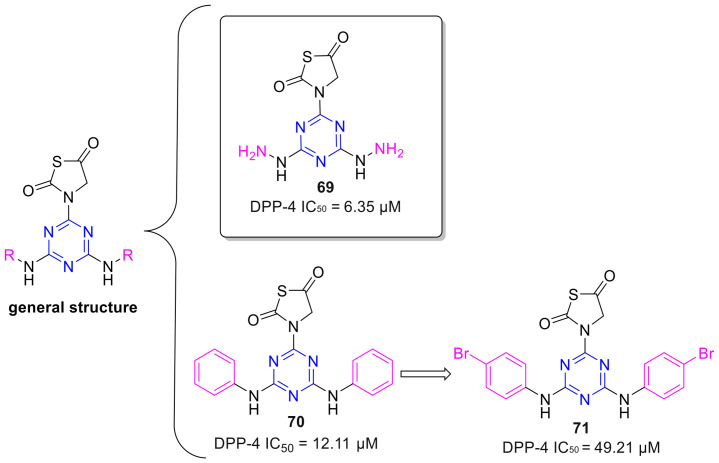
Structures of selected compounds, as described by Srivastava et al. [83].

Srivastava et al. [83] described **eleven** novel 1,3,5-triazino-thiazolidino-2,4-diones as DPP-4 inhibitors with antibacterial activity for the treatment of T2D (Figure 33). Such dual activity can be useful, as infections are frequently contracted by diabetics. Among the synthesised series of hybrid 1,3,5-triazino–thiazolidine-2,4-diones, compounds **69**–**70** attracted particular attention because they represented different structural characteristics and displayed significantly different biological activities toward the DPP-4 enzyme.

Compound **69** contained a hydrazine substituent, which proved to be the most favourable group for DPP-4 inhibitory activity. This derivative exhibited the highest activity in the entire series with an IC_50_ value of 6.35 µM. The authors suggested that the relatively small and more hydrophilic nature of the substituent facilitated efficient accommodation within the enzyme’s S1 pocket. Molecular docking studies demonstrated that the thiazolidine-2,4-dione ring of compound **69** was oriented toward the hydrophobic S1 pocket formed by residues Tyr631, Val656, Trp659, Tyr662, Tyr666, and Val711. One amino group formed a hydrogen bond with Glu205, an important residue of the *N*-terminal recognition region, while the second amino group interacted with Tyr547. Additionally, a π–cation interaction with Arg125 was observed. The high CDOCKER interaction energy (31.90) and CDOCKER energy (44.31) indicated stable binding of the ligand within the active site of DPP-4. Introduction of the molecule phenyl (compound **70**; Figure 33) or substituted phenyl moiety (e.g., compound **71**; Figure 33) caused a decrease in activity in comparison with compound **69** (Figure 33). Compound **70** showed about two-fold decrease in this activity (IC_50_ of 12.11 µM), whereas the *para*-bromophenyl-substituted compound **71** displayed the weakest activity in the series with an IC_50_ value of 49.21 µM. SAR analysis showed that hydrophobicity and increased steric size of the substituents led to a significant loss of biological activity. Antibacterial activity of compounds was evaluated using the broth microdilution method against Gram-positive bacteria (*Bacillus subtilis*, *Bacillus cereus*, and *Staphylococcus aureus*) and Gram-negative bacteria (*Escherichia coli*, *Proteus vulgaris*, and *Pseudomonas aeruginosa*). Cefixime was used as the reference antibacterial agent. In the tests carried out, the compounds exhibited moderate or very good activity against the tested microorganisms. The level of activity depended on the strain tested. Compound **69** exhibited one of the weakest antibacterial activities in the entire series.

#### 5.9.2. 1,3,5-Triazines with Inhibitory Activity for α-Glucosidase, Carbonic Anhydrase, and Acetylcholinesterase

**Figure 34:**
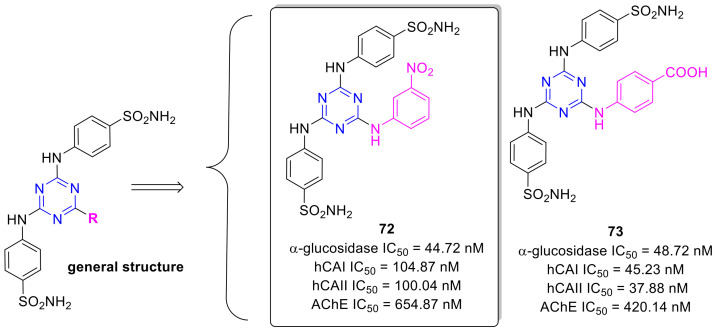
Structures of the most potent multi-target compounds, as described by Lolak et al. [84]. hCAI, human carbonic anhydrase I; hACII, human carbonic anhydrase II; AChE, acetylcholinesterase.

Lolak et al. [84] presented syntheses, biological studies, and modelling for a series of **eleven** compounds. The compounds were designed based on previous studies to target three biological targets: carbonic anhydrase, acetylcholinesterase, and α-glucosidase. The authors indicated that such a multi-target effect on these selected targets could be beneficial in the treatment of many diseases, including diabetes. Carbonic anhydrase inhibitors are involved in the pathogenesis and development of many diseases, including cardiovascular and cardiometabolic diseases [85]. The general structures of these compounds are shown in Figure 34. α-Glucosidase inhibitory activity was determined by spectrofluorometry using *para*-nitrophenyl-D-glucosidase as a substrate. All compounds exhibited inhibitory activity against this enzyme with IC_50_s < 100 μM. This activity was higher than that of the reference inhibitor, acarbose (IC_50_ = 118 nM). The highest activity was exhibited by compound **72** (IC_50_ = 44.72 nM; Figure 34). In contrast, compound **73** (Figure 31) exhibited the highest multi-target activity. Docking studies were performed against all biological targets for the most potent compounds. For compound **72** to α-glucosidase (PDB:5NN8), revealed hydrogen bonding with ASp282 and Asp518, as well as π–π interactions with Trp481 at the α-glucosidase binding site.

#### 5.9.3. 1,3,5-Triazines with Anti-Inflammatory and Antidiabetic Activities

**Figure 35:**
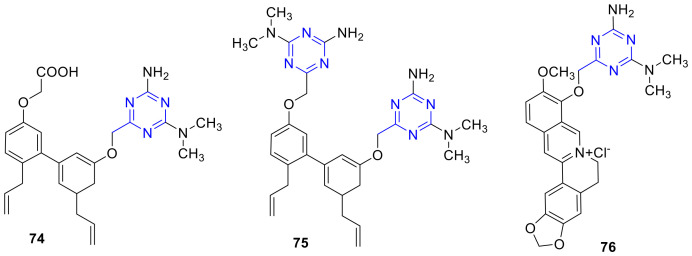
Structures of compounds, as described by Cao et al. [86].

Cao et al. [86] described the synthesis and in vitro studies of three compounds derived from two alkaloids, magnolol and berberine, as triazine derivatives. Both of these alkaloids have been used in traditional Chinese medicine to treat, amongst other conditions, diabetes. Cyclisation with metformin resulted in three derivatives, with the structures shown in Figure 35. Biological studies were conducted on RAW264.1 (anti-inflammatory) and INS-1 (antidiabetic) cell lines using the MTT assay. In LPS-stimulated RAW264.1 cells, all compounds showed a reduction in COX2 levels ranging from 13.1% (compound **74**), 14.2% (compound **75**), to 25.4% (compound **76**), and PEG2 from 27.1% (compound **69**), 32.3% (compound **75**), to 36.4% (compound **76**). In INS-1 cells, the compounds caused an increase in glucose-mediated (16.7 mM) insulin secretion by 137% (compound **74**), 156% (compound **75**), and 143% (compound **76**).

## 6. Discussion

Type 2 diabetes mellitus is a complex metabolic disorder characterised by chronic hyperglycaemia resulting from impaired insulin secretion, insulin resistance, and dysregulated energy metabolism. In addition to disturbances in glucose homeostasis, the disease is strongly associated with oxidative stress, chronic inflammation, and activation of alternative metabolic pathways, which collectively contribute to the development of long-term complications. Current therapeutic strategies target multiple mechanisms involved in glucose regulation. Inhibition of digestive enzymes, such as α-glucosidase and α-amylase, reduces postprandial hyperglycaemia by delaying carbohydrate digestion, whereas inhibition of DPP-4 enhances incretin signalling and insulin secretion. Other approaches include modulation of the polyol pathway through inhibition of aldose reductase and sorbitol dehydrogenase, as well as suppression of AGE formation and AGE–RAGE-mediated inflammation.

Their chemical structure enables modulation of enzyme binding, redox balance, and inflammatory signalling, making them promising candidates for multifunctional antidiabetic therapy. The evaluation of such compounds relies on an integrated approach combining in vitro biochemical assays, cell-based studies, and in vivo models, each providing complementary information. Enzymatic assays constitute the first step of screening and are used to confirm target engagement. These assays allow rapid identification of active compounds and provide mechanistic insight into enzyme–ligand interactions. However, recently preliminary in silico studies are also used to increase the possibility of success and minimise biological studies.

Subsequently, cell-based assays are employed to assess cytotoxicity and biological activity in a more complex system. Viability tests, such as MTT or WST-1, measure mitochondrial metabolic activity and are used to determine whether compounds exhibit toxic effects at biologically relevant concentrations. A panel of cell lines is typically used to obtain a broad safety profile. Rapidly proliferating cancer cell lines, such as HCT-116, MDA-MB-231, and A549, are particularly sensitive to mitochondrial dysfunction and are therefore useful for detecting general cytotoxicity, while metabolically relevant models, such as HepG2 hepatocytes, allow assessment of hepatic responses. In parallel, THP-1 monocytes are employed to investigate inflammatory processes relevant to diabetes, including activation of the AGE–RAGE axis.

To further elucidate mechanisms of action, specialised biochemical and molecular assays are applied. ROS detection assays are used to evaluate intracellular oxidative stress, which plays a central role in insulin resistance and diabetic complications. Western blot analysis enables quantification of specific proteins and their activation states, particularly phosphorylation-dependent signalling pathways, such as NF-κB and MAPK, which regulate inflammation and cellular stress responses. Immunocytochemistry provides complementary spatial information by visualising subcellular localization of key regulators, such as nuclear translocation of transcription factors. In addition, ELISA assays allow sensitive quantification of secreted inflammatory mediators, reflecting the functional outcome of pathway modulation at the extracellular level. Fluorescence-based antiglycation assays further assess the ability of compounds to inhibit AGE formation, linking molecular activity with the prevention of diabetes-associated tissue damage.

Finally, in vivo models are essential for validating pharmacological efficacy under physiological conditions. Chemically induced diabetic models, such as alloxan- or streptozotocin-treated rodents, are widely used to evaluate antihyperglycaemic activity, lipid metabolism, and progression of complications. Zebrafish are also used as a model organism in metabolic disorder research because of their genetic resemblance to humans, inexpensive breeding, and compatibility with high-throughput screening methods [87].

In this review, the analysis covered 30 publications (excluding those concerning fotagliptin and imeglimin) that described triazine derivatives with various substituents.

A large group here consists of 1,2,4-triazines, which occurred as single rings or in fused systems. In fused rings, the 1,2,4-triazine ring was fused to indole or 1,2,4-triazole.

The compounds described exhibited various levels of activity depending on the biological target. Their activity was compared to that of a reference compound for the particular biological target. Typically, the best compounds exhibited activity comparable to or better than that of the reference compounds. In vitro tests used to assess antidiabetic activity can be divided into two main types: target-based screens and phenotypic screens [88]. In the studies analysed, a single type of test from the target-based screens was typically used, although it would certainly be better to also carry out tests from the phenotypic group in order to gain a better understanding of how these compounds work. In some cases, additional studies were conducted to assess the toxicity of these compounds or the selectivity of their action.

Over the past 25 years, two drugs, fotagliptin and imeglimin, have been introduced into clinical practice, although so far only in the markets of the countries where they were approved. Several compounds (ENOblock—CAS1177827-73-4, CP-642931—CAS300551-49-9, cemtirestat—CAS309283-89-4), despite not reaching clinical trials, are commercially available and constitute valuable tools in in vitro and in vivo studies. Triazines, therefore, constitute a biological system with great potential for structural modification, reflected in the diversity of observed pharmacological effects. Table 1 summarises the articles discussed (without those connected with fotagliptin and imeglimin) and presents the most important information.

## 7. Conclusions

Type 2 diabetes is a common metabolic disorder associated with insulin resistance and serious long-term complications. Recent studies suggest that triazine derivatives are promising antidiabetic agents that may regulate glucose metabolism and prevent diabetes-related complications. Over the past 25 years, numerous triazines have been synthesised. Typically, compounds were rationally designed based on the results of previous studies. Their activity was compared with that of known drugs or active compounds targeting relevant biological targets. A large group of derivatives consists of compounds containing a 1,2,4-triazine ring, often fused with another heterocyclic system, such as indole or 1,2,4-triazole. In contrast, 1,3,5-triazine derivatives do not occur in fused systems, whilst 1,2,3-triazines were the least frequently used in the search for antidiabetic compounds. The most common substituents introduced into the molecules were halogens (F, Cl), the nitro group, the methoxy group, or the methyl group. A combination of enzymatic, cellular, and animal studies provided a comprehensive framework for the identification and characterisation of new triazines. For most compounds, in vitro studies were limited to a single pharmacological target. Their ADMET parameters were not investigated. In contrast, in a large number of studies, molecular docking was carried out to elucidate the mechanism of action. Two compounds (fotagliptin and imeglimin) were launched on the market in some countries, but not in Europe or the United States. Thus, triazines represent a promising class of compounds for the further development as antidiabetic drugs.

## Figures and Tables

**Table 1:** Summary of described triazines as antidiabetic agents (without fotagliptin and imeglimin).

No	Reference	Isomer				Number of Described Compounds	Type of Studies		Biological Activity Range	Other Studies
		Fused 1,2,3- Triazine	1,2,4-Triazine	Fused 1,2,4-Triazine	1,3,5-Triazine		In Vitro	In Vivo (or Ex Vivo)		
1	Khalid et al. [20]	Yes	-	-	-	**13**	Yes		α-Glucosidase inhibitors IC_50_: 29.8–102.2 μM	Molecular docking (UniProt:P53051)
2	Wang et al. [21]	-	Yes	-	-	**17**	Yes	-	α-Glucosidase inhibitors IC_50_: 12.5–72.7 μM	Molecular docking (PBD:3AJ7)
3	Valipour et al. [22]	-	Yes	-	-	**10**	Yes	Yes	α-Glucosidase inhibitors IC_50_: 12.0–672.6 μM	Molecular docking (PDB:7P2Z) Enzyme kinetics Cytotoxicity (lines: HCT-116, MDA-MB-231, A549) in MTT assay
4	Jahan et al. [23]	-	Yes	-	-	**26**	Yes	-	Glycation inhibitors IC_50_: 91.0–259.0 μM	MTT assay, WST-1 assay, Western blot, immunocytochemistry
5	Roney et al. [28]	-	Yes	-	-	**12**	-	-	Aldose reductase inhibitors (not confirmed—only in silico studies)	Molecular docking (PBD:1US0), MD simulations (200 ns) MM/GBSA, MD, PCA
6	Kang et al. [32]	-	Yes	-	-	**2**	Yes	-	SGLT2 inhibitors IC_50_ = 24.9 nM & IC_50_ = 491 nM	---
7	Chen et al. [43]	-	Yes	-	-	**41**	Yes	Yes	GLP-1R agonists EC_50_: 6 pM to >10,000 nM	hERG channel inhibition Molecular docking (PDB ID:6X1A) Pharmacokinetic studies in rats for compound **21:** t_1/2_ = 1.1 h; C_max_ = 130 ng/mL AUC_last_ = 70 h·ng/mL In vivo (in human GLP-1R Knock-In mice): —oral glucose tolerance test —food intake test
8	Shamim et al. [26]	-	Yes	-	-	**24**	Yes	-	α-Amylase & α-glucosidase inhibitors IC_50_ < 50 μM IC_50_: 13.0–46.9 µM (α-amylase) IC_50_: 13.1–46.4 µM (α-glucosidase)	Molecular docking (PDB:3AJ7, α-glucosidase) (PDB:3BAJ, α-amylase) Kinetic studies
9	Khan et al. [47]	-	Yes	-	-	**12**	Yes	-	Multi-target ligands (α-amylase, anticancer & antiviral) IC_50_: 3.10–25.80 μM (α-amylase) IC_50_: 0.20–19.10 μM (anticancer), IC_50_: 3.20–16.20 μM (SARS-CoV-2)	Molecular docking (PBD:6LU7, SARS-CoV-2)
10	Seyfi et al. [48]	-	-	Yes	-	**15**	Yes	-	Dual α-amylase & α-glucosidase inhibitors IC_50_: 24.64–115.57 nM (α-amylase) IC_50_: 34.52–213.44 nM (α-glucosidase)	Molecular docking (PDB—no precise information) Kinetic studies
11	Patel et al. [49]	-	-	Yes	-	**17**	Yes	Yes	DPP-4 inhibitors (only 2 active) IC_50_ = 28.1 μM & IC_50_ = 166.4 μM	Molecular docking (PBD:3KWF) Oral glucose tolerance tests A chronic model of high-fat diet fed with streptozotocin in rats
12	Guertin et al. [50]	-	-	Yes	-	**15**	Yes	Yes	PTP1B inhibitors IC_50_ (300 nM dithiothreitol): 2.9 to >100 μM	Pharmacokinetic studies in C57BL/6J mice for compound **33:** t_1/2_ = 1.1 h; CL = 104 mL/Kg/min V_ss_ = 3.1 L/kg; C_max_ = 4.0 μM F = 97% An ob/ob mouse model
13	Rahim et al. [52]	-	-	Yes	-	**11**	Yes	-	α-Glucosidase inhibitors IC_50_: 2.3–312.8 μM	Molecular docking (PBD:2AJ7)
14	Taha et al. [53]	-	-	Yes	-	**25**	Yes	-	α-Glucosidase inhibitors IC_50_: 1.3–5.8 µM	Molecular docking (PBD:3W37)
15	Rahim et al. [54]	-	-	Yes	-	**21**	Yes	-	α-Amylase inhibitors IC_50_: 1.2–21.50 µM	Molecular docking (PBD:1OSE)
16	Aggarwal et al. [55]	-	-	Yes	-	**10**	Yes	-	α-Amylase inhibitors IC_50_: 16.14–27.69 μg/mL (IC_50_: 41.7–75.6 µM)	Molecular docking (PBD:7TAA)
17	Stefek et al. [57]	-	-	Yes	-	**15**	Yes	Yes (ex vivo)	Aldose reductase inhibitors ALR2 IC_50_: 0.097–53.5 µM ALR1 IC_50_: 1.2–80.2 µM	Crystal structure AKR1B10 inhibition Enzyme kinetics Sorbitol accumulation in isolated rat eye lenses cultivated with glucose (50 mM)
18	Hlaváč et al. [62]	-	-	Yes	-	**4**	Yes	Yes (ex vivo)	Aldose reductase inhibitors ALR2 IC_50_: 51–787 nM (1% DMSO) ALR2 IC_50_: 42–434 nM (H_2_O)	AKR1B1 inhibition AKR1B10 inhibition Sorbitol accumulation in isolated rat eye lenses cultivated with glucose (50 mM) MD simulations
19	Khodakhah et al. [69]	-	-	-	Yes	**10**	-	Yes	Antihyperglycaemic agents Fasting blood sugar of zebrafish diabetic model: 72.3–108.3 mg/dL	Molecular docking SIRT1 (PBD:5BTR) and GSK-3β (PBD:1Q4L) Zebrafish diabetic model
20	Ahmadi et al. [71]	-	-	-	Yes	**2**	-	Yes	Antihyperglycaemic agents Antihyperlipidaemic agents	Alloxan-induced diabetic rat model
21	Andrews et al. [72]	-	-	-	Yes	**16**	Yes		DPP-4 inhibitors IC_50_: 1.6–1400 nM	Many in vitro assays
22	Gao et al. [73]	-	-	-	Yes	-	Yes	Yes	DPP-4 inhibitors IC_50_: 2.3–544.4 nM	DPP-8 inhibition, DPP-9 inhibition Molecular docking (PBD:2FJP) Oral glucose tolerance test (male IRC mice) STZ-induced diabetes in rats
23	Cho et al. [74]	-	-	-	Yes	**1** (ENOblock)	Yes	Yes	Glycolytic enzyme enolase modulator IC_50_ = 576 nM	Gene expression analysis and histology Diabetic db/db mice
24	Mylari et al. [76]	-	-	-	Yes	**10**	Yes	Yes	Sorbitol dehydrogenase inhibitors IC_50_: 5–39 nM (rat) IC_50_: 4–43 nM (human)	Pharmacokinetic studies (compound **56** serum half-lives: 7 h—rats; 10 h—dogs) Two streptozotocin diabetic rat models (acute and chronic)
25	Mylari et al. [80]	-	-	-	Yes	**10**	Yes	Yes	Sorbitol dehydrogenase inhibitors IC_50_: 36–400 nM (rat) IC_50_: 42–390 nM (human)	Two streptozotocin diabetic rat models (acute and chronic)
26	El-Harakeh et al. [81]	-	-	-	Yes	**8**	Yes	-	Antidiabetic nephropathy agent	MTT assay and Western blot
27	Jung et al. [82]	-	-	-	Yes	**6**	Yes	-	Insulin mimetic agents	Cytotoxicity and anti-inflammatory studies
28	Srivastava et al. [83]	-	-	-	Yes	**11**	Yes	-	DPP-4 inhibitors IC_50_: 6.4–49.2 μM & antibacterial activity	Molecular docking (PBD:2FJP)
29	Lolak et al. [84]	-	-	-	Yes	**11**	Yes	-	α-Glycosidase inhibitors (IC_50_ = 44.7–84.3 μM) Acetylcholinesterase inhibitors (IC_50_: 397.3–856.3 μM) Carbonic anhydrase inhibitors IC_50_: 45.2–207.2 μM (CA I) IC_50_: 37.8–194.5 μM (CA II)	Molecular docking (PBD:5NN8, α-glycosidase) (PBD:4M0F, acetylcholinesterase) (PBD:4WUQ, CA I) (PBD:4FU5, CA II)
30	Cao et al. [86]	-	-	-	Yes	**3**	Yes	-	Antidiabetic (INS-1 cells) & anti-inflammatory (RAW264.1 cells)	----

## Data Availability

No new data were created or analysed in this study. Data sharing is not applicable to this article.

## Abbreviations

The following abbreviations are used in this manuscript:

3T3-L1	Mouse 3T3 Fibroblast-Derived Adipocyte Cell Line
6-NBDG	6-(*N*-(7-Nitrobenz-2-Oxa-1,3-Diazol-4-Yl)Amino)-6-Deoxyglucose
ADME	Absorption, Distribution, Metabolism, and Excretion
AGE	Advanced Glycation End Product
AKR1B1	Aldo-Keto Reductase Family 1 Member B1
ALR	Aldose Reductase
ALR1	Aldehyde Reductase 1
ALR2	Aldose Reductase 2
AMPK	AMP-Activated Protein Kinase
APC	Article Processing Charge
AR	Aldose Reductase
ATP	Adenosine Triphosphate
ATPase	Adenosine Triphosphatase
CA	Carbonic Anhydrase
Caco-2	Human Epithelial Colorectal Adenocarcinoma Cell Line
CHO	Chinese Hamster Ovary
CONSORT	Consolidated Standards of Reporting Trials
COX-2	Cyclooxygenase-2
CRP	C-Reactive Protein
DCFH-DA	2,7-Dichlorodihydrofluorescein Diacetate
DMSO	Dimethyl Sulfoxide
DPP-4	Dipeptidyl Peptidase-4
DPP-8	Dipeptidyl Peptidase-8
DPP-9	Dipeptidyl Peptidase-9
ELISA	Enzyme-Linked Immunosorbent Assay
FDA	Food and Drug Administration
FBS	Fasting Blood Sugar
FFA	Free Fatty Acid
GIP	Glucose-Dependent Insulinotropic Polypeptide
GLP-1	Glucagon-Like Peptide-1
GLUT1	Glucose Transporter 1
GLUT2	Glucose Transporter 2
GLUT4	Glucose Transporter 4
GSIS	Glucose-Stimulated Insulin Secretion
GSK-3β	Glycogen Synthase Kinase-3 Beta
hALR	Human Aldose Reductase
HbA1c	Glycated Haemoglobin
HDAC4	Histone Deacetylase 4
HDL	High-Density Lipoprotein
HG	High Glucose
IL-6	Interleukin-6
LDL	Low-Density Lipoprotein
MAPK	Mitogen-Activated Protein Kinase
MDA-MB-231	Human Breast Cancer Cell Line
MD	Molecular Dynamics
MGO	Methylglyoxal
MM/GBSA	Molecular Mechanics/Generalised Born Surface Area
MTT	3-(4,5-Dimethylthiazol-2-Yl)-2,5-Diphenyltetrazolium Bromide
NAD+	Nicotinamide Adenine Dinucleotide
NADPH	Nicotinamide Adenine Dinucleotide Phosphate
NEFA	Non-Esterified Fatty Acid
NF-κB	Nuclear Factor Kappa B
PDB	Protein Data Bank
PGE2	Prostaglandin E2
PPARγ	Peroxisome Proliferator-Activated Receptor Gamma
PPH	Postprandial Hyperglycaemia
RAGE	Receptor for Advanced Glycation End Products
ROS	Reactive Oxygen Species
SAA	Serum Amyloid A
SAR	Structure–Activity Relationship
SDH	Sorbitol Dehydrogenase
SGLT1	Sodium–Glucose Cotransporter 1
SGLT2	Sodium–Glucose Cotransporter 2
SIRT1	Sirtuin 1
T2D	Type 2 Diabetes
THP-1	Human Monocytic Cell Line THP-1
TIMES	Trials of Imeglimin for Efficacy and Safety
TNF-α	Tumour Necrosis Factor Alpha
TZD	Thiazolidinedione
VCAM-1	Vascular Cell Adhesion Molecule 1
WST-1	Water-Soluble Tetrazolium Salt-1 Assay
ZDF	Zucker Diabetic Fatty
